# Soft surfaces promote astrocytic differentiation of mouse embryonic neural stem cells via dephosphorylation of MRLC in the absence of serum

**DOI:** 10.1038/s41598-021-99059-5

**Published:** 2021-10-01

**Authors:** Hiroshi Oyama, Akihiro Nukuda, Seiichiro Ishihara, Hisashi Haga

**Affiliations:** 1 Business Development, Shionogi & Co. Ltd., 1-8, Doshomachi 3-chome, Chuo-ku, Osaka, 541-0045 Japan; 2 Division of Life Science, Graduate School of Life Science, Hokkaido University, N10-W8, Kita-ku, Sapporo, 060-0810 Japan; 3 Department of Advanced Transdisciplinary Sciences, Faculty of Advanced Life Science, Hokkaido University, N10-W8, Kita-ku, Sapporo, 060-0810 Japan

**Keywords:** Cell biology, Stem cells

## Abstract

Astrocytes, which can be obtained from neural stem cells (NSCs) by adding serum and/or recombinant proteins in culture media or by passaging NSCs repeatedly, are expected to be applicable in regenerative medicine for the treatment of neurodegenerative diseases. However, astrocytes obtained using existing methods are costly and have poor quality. The stiffness of culture surfaces has been reported to affect astrocytic differentiation of adult NSCs. However, the influence of surface stiffness on astrocytic differentiation of embryonic NSCs has not yet been reported. In this study, we showed that astrocytic differentiation of embryonic NSCs was increased on soft surfaces (1 kPa and 12 kPa) compared with the NSCs on stiff surfaces (2.8 GPa) in serum-free condition. Furthermore, di-phosphorylated myosin regulatory light chain (PP-MRLC) was decreased in embryonic NSCs cultured on the soft surfaces than the cells on the stiff surfaces. Additionally, astrocytic differentiation of embryonic NSCs was induced by a Ras homolog associated kinase (ROCK) inhibitor, which decreased PP-MRLC in NSCs. These results suggest that decreasing the PP-MRLC of embryonic NSCs on soft surfaces or treating NSCs with a ROCK inhibitor is a good method to prepare astrocytes for application in regenerative medicine.

## Introduction

Neural stem cells (NSCs) can differentiate into neurons and glia. Among the glia, astrocytes are expected to be applicable in regenerative medicine for neurodegenerative diseases, such as amyotrophic lateral sclerosis (ALS) and spinal cord injury (SCI)^1–4^. Astrocytes can be obtained from NSCs by culturing NSCs in a medium containing serum and/or recombinant proteins to trigger astrocytic differentiation or by passaging NSCs repeatedly^5–8^. However, these existing methods are not ideal for regenerative medicine applications. For example, serum contains many undefined proteins, including unknown pathogens^9^. In addition, the repeated use of recombinant proteins or passaging NSCs is costly and time-consuming. Therefore, low-cost and fast methods for preparing astrocytes from NSCs under pathogen-free conditions are strongly required.

Two types of NSCs can be obtained from the mammalian brain: (1) embryonic NSCs isolated from the ganglionic eminence of the embryonic brain^10^, and (2) adult NSCs, isolated from the ganglionic eminence of the fetal brain or subventricular zone of the lateral ventricles and the subgranular layer of the hippocampus of the adult brain^11^. There are human NSCs in the brain; however, we cannot isolate them from the brain by surgery for regenerative medicine. Alternatively, human NSCs can be induced from human induced pluripotent stem (iPS) cells for regenerative medicine. However, the human iPS cell-derived NSCs have embryonic characteristics and find it difficult to differentiate into astrocytes; hence, it takes over 10 passages/80 days of human NSCs to prepare for differentiation and 14 days of prepared NSCs to differentiate into human astrocytes in the culture medium containing serum^12^. Therefore, easier and faster methods for differentiating astrocytes from embryonic and neurogenic NSCs are strongly required for regenerative medicine for neurodegenerative diseases, such as ALS and SCI.

Differentiation of various types of cells is strongly influenced by culture surface stiffness^13–17^. It has been reported that stiffness of culture surfaces affects the differentiation of adult NSCs^18–20^. Adult NSCs tend to differentiate into neurons on soft culture surfaces, whereas they easily differentiate into astrocytes on stiff culture surfaces regardless of the presence or absence of serum. On stiff surfaces, embryonic NSCs preferably differentiate into neurons in serum-free media, whereas in a culture medium containing serum, they easily differentiate to astrocytes^21^. However, there are no reports that soft surfaces regulate the differentiation of embryonic NSCs.

It has been shown that actomyosin—a cytoskeletal complex that generates mechanical contractile force in cells—is involved in the regulation of cell fate and differentiation^22,23^. Actomyosin contractility is upregulated by the di-phosphorylation of myosin regulatory light chain (MRLC). Di-phosphorylation of MRLC (PP-MRLC) is increased in the cells on stiff surfaces, and as a result, enhances the cellular contractile force^24,25^. Conversely, cellular contractile force is loosened by MRLC dephosphorylation in cells on soft surfaces. Di-phosphorylation of MRLC is known to be enhanced by the activation of the Ras homolog family member A (RhoA) signaling cascade^26^. It was reported that the inhibitor of Rho-associated kinase (ROCK)—a key protein in the RhoA signaling cascade—prevents the phosphorylation of MRLC in various types of cells and changes the morphology of the cells on stiff surfaces^24,27–30^. However, whether PP-MRLC in NSCs is regulated by surface stiffness, and whether it directs the differentiation of NSCs is not well understood.

In this study, we examined whether astrocytic differentiation of embryonic NSCs was influenced by surface stiffness in the absence of serum. The results showed that differentiation of embryonic NSCs to astrocytes was significantly increased on soft surfaces compared with commonly used stiff plastic surfaces. In addition, the expression of PP-MRLC in embryonic NSCs cultured on stiff surfaces was higher than that in cells cultured on soft surfaces. Y27632, a widely used ROCK inhibitor, reduced PP-MRLC in embryonic NSCs and promoted astrocytic differentiation of embryonic NSCs on stiff surfaces. These results suggest that in serum-free conditions, culturing embryonic NSCs on soft surfaces or with Y27632 might be a suitable alternative to prepare astrocytes in regenerative medicine.

## Results

### Soft culture surfaces promote the differentiation of embryonic NSCs into astrocytes in the absence of serum

**Figure 1:**
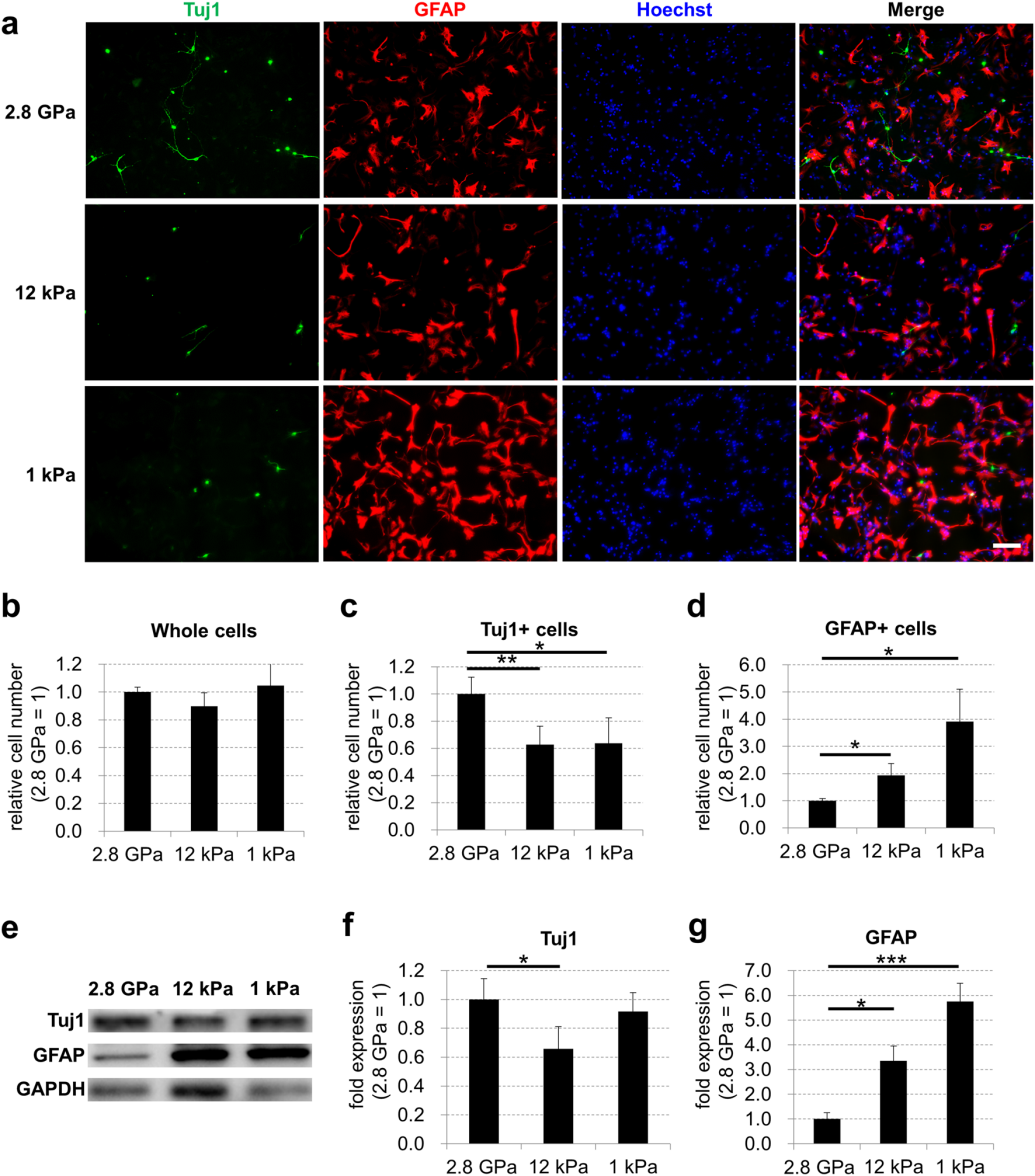
Soft surfaces increase astrocytes differentiated from mouse embryonic neural stem cells in the absence of serum. (**a**) Mouse embryonic NSCs were cultured on three types of plate (1 kPa plates, 12 kPa plates, and commonly used plastic plates [2.8 GPa]) for 3 days in serum-free condition and assessed by immunofluorescence staining. Neurons were visualized by Tuj1 (green), astrocytes were visualized by GFAP (red), and cell nuclei were counterstained with Hoechst (blue). Scale bar, 100 μm. (**b**) Whole cell number, (**c**) the number of Tuj1 positive cells, and (**d**) the number of GFAP positive cells on each plate were counted and normalized relative to the control. (**e**) Protein expression of Tuj1, GFAP, and GAPDH (for loading control) on each plate was detected by western blotting analysis. The expression levels of (**f**) Tuj1 and (**g**) GFAP proteins were normalized relative to GAPDH protein. Error bars represent the standard deviation; *p < 0.05, **p < 0.01, ***p < 0.005 (Student’s *t* test with Bonferroni correction). *NSCs* neural stem cells, *GFAP* glial fibrillary acidic protein, *GAPDH* glyceraldehyde 3-phosphate dehydrogenase.

**Figure 2:**
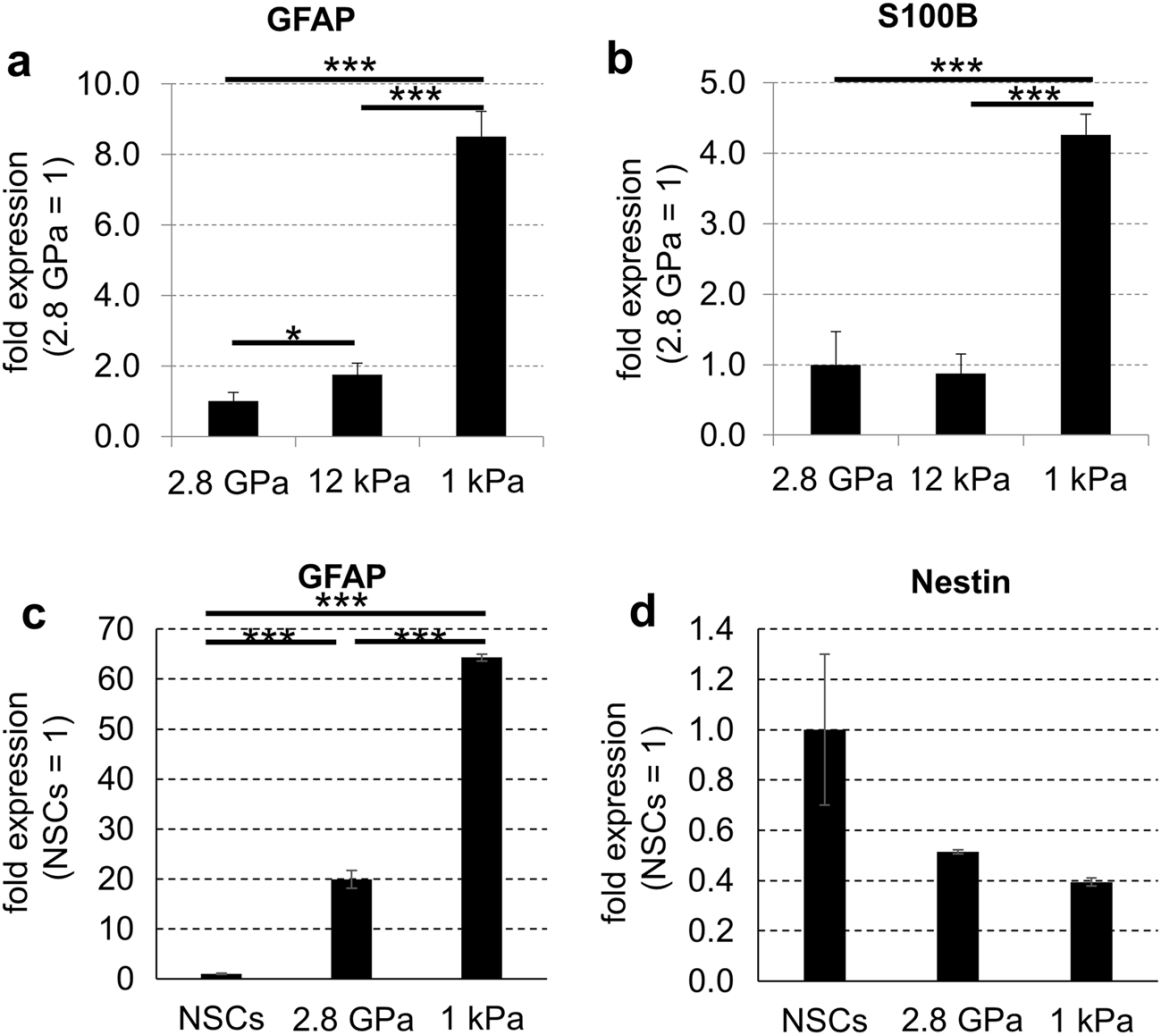
Soft surfaces increase mRNA expression of astrocyte markers and decrease mRNA expression of NSCs marker after 3 days of differentiation of mouse embryonic neural stem cells in the absence of serum. Mouse embryonic NSCs were cultured on three types of plates (1 kPa plates, 12 kPa plates, and commonly used plastic plates [2.8 GPa]) for 3 days in serum-free conditions. The mRNA expression of GFAP, S100B, and GAPDH on each plate type was detected by qPCR analysis. The expression levels of (**a**) GFAP and (**b**) S100B were normalized relative to that of GAPDH. Mouse embryonic NSCs were cultured on two types of plates (1 kPa plates and commonly used plastic plates [2.8 GPa]) for 24 h in serum-free conditions. The mRNA expression of GFAP, Nestin, and GAPDH on each plate type and pre-seeding NSCs were detected by qPCR analysis. The expression levels of (**c**) GFAP and (**d**) Nestin were normalized relative to that of GAPDH. Error bars represent the standard deviation; *p < 0.05, ***p < 0.005 (Student’s *t* test with Bonferroni correction). *NSCs* neural stem cells, *GFAP* glial fibrillary acidic protein, *S100B* S100 calcium binding protein B, *GAPDH* glyceraldehyde 3-phosphate dehydrogenase, *qPCR* quantitative polymerase chain reaction.

To examine the influence of culture surface stiffness on astrocytic differentiation of embryonic NSCs in the absence of serum, we obtained neurogenic NSCs from embryonic day (E) 11.5 mouse embryonic forebrain and cultured the cells on three types of plate with different stiffness (1 kPa plates, 12 kPa plates, and commonly used stiff plastic plates [2.8 GPa]) for 3 days with a serum-free medium supplemented with B27. Immunostaining showed that the number of whole cells was not significantly different among the cells on these plates, whereas the number of neuronal marker Tuj1 (neuron-specific class III beta-tubulin)-positive cells was decreased significantly in the cells on the soft surfaces compared with the cells on the stiff surfaces (Fig. 1a–c). In addition, the number of glial fibrillary acidic protein (GFAP)-positive cells was significantly increased in the cells on the soft surface plates compared with the stiff surfaces (Fig. 1a,d). Western blotting also revealed that the protein expression of Tuj1 was higher in the cells on a plastic plate than in the cells on surfaces at 12 kPa (Fig. 1e,f). In contrast, GFAP protein expression in the cells on soft surfaces was higher than that on stiff surfaces (Fig. 1e,g and Supplementary Fig. S1). Furthermore, qPCR analysis revealed that the mRNA expression of GFAP and mature astrocyte marker S100 calcium-binding protein B (S100B) was higher in the cells on soft surfaces than those on stiff surfaces (Fig. 2a–c). In addition, the mRNA expression of Nestin, a marker of undifferentiated NSCs, decreased after seeding on each plate (Fig. 2d). These results indicate that soft culture surfaces prevent the differentiation of embryonic NSCs into neurons and promote the differentiation of NSCs into astrocytes in the absence of serum.

### Soft surfaces decrease PP-MRLC in NSCs

**Figure 3:**
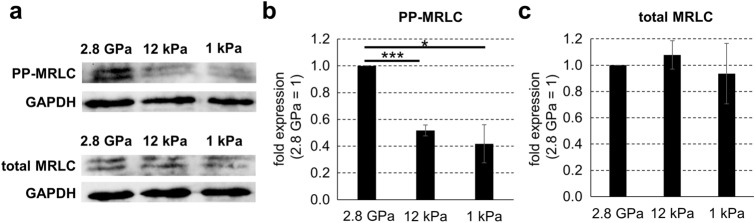
PP-MRLC of mouse embryonic neural stem cells is decreased by culturing on soft surfaces in the absence of serum. (**a**) Mouse embryonic NSCs were cultured on three types of plate (1 kPa plates, 12 kPa plates, and commonly used plastic plates [2.8 GPa]) for 3 days in serum-free condition. Protein expression of PP-MRLC, total MRLC, and GAPDH (for loading control) on each plate type was detected by western blotting analysis. The expression levels of (**b**) PP-MRLC and (**c**) total MRLC proteins were normalized relative to that of GAPDH protein. Error bars represent the standard deviation; *p < 0.05, ***p < 0.005 (Student’s *t* test with Bonferroni correction). *NSCs* neural stem cells, *PP-MRLC* di-phosphorylated myosin regulatory light chain.

It has been reported that actomyosin contractility generates mechanical stress in the cells, and as a result, regulates cell fate decision and differentiation^22,23^. The mechanical stress generated by actomyosin contractility was enhanced by the di-phosphorylation of MRLC. We reported that stiff surfaces increased PP-MRLC, which enhanced cellular contractile force^24,25^. Therefore, we examined whether surface stiffness regulates the expression of PP-MRLC in NSCs. We cultured mouse embryonic NSCs for 3 days on plates with different stiffness (1 kPa plates, 12 kPa plates, and commonly used stiff plastic plates [2.8 GPa]) in serum-free conditions and examined the expression of PP-MRLC and total MRLC. Western blotting revealed that PP-MRLC was significantly decreased in cells on the soft surfaces compared with the cells on the stiff surfaces, although there was no significant difference in the expression of total MRLC among the cells on these plates (Fig. 3a–c). These results indicate that soft surfaces reduce the expression of PP-MRLC in NSCs.

### Treatment of a ROCK inhibitor reduces PP-MRLC in NSCs

**Figure 4:**
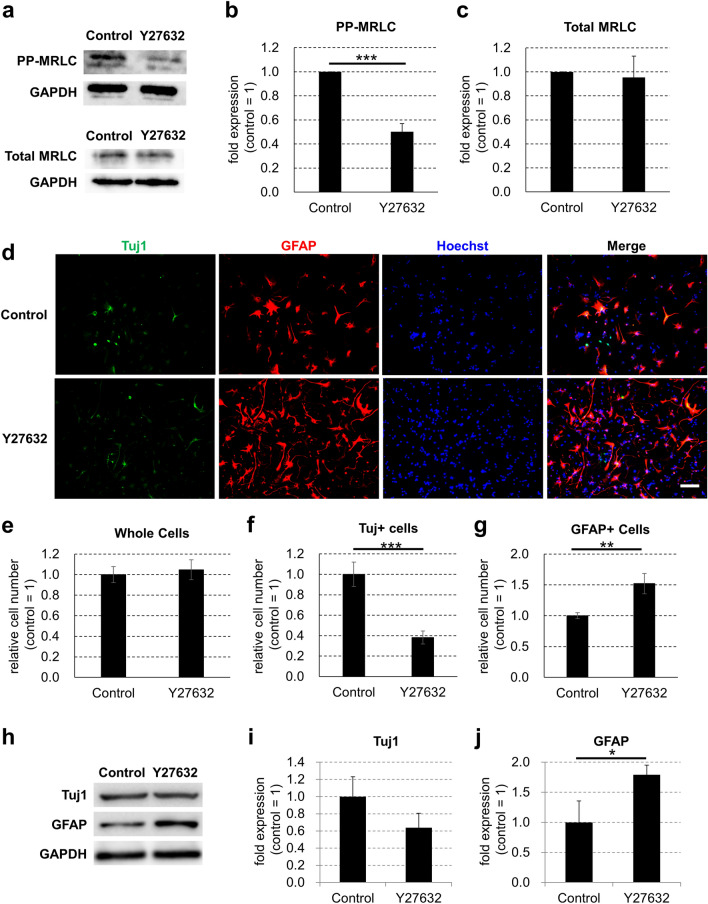
Treatment of ROCK inhibitor decreases PP-MRLC and increases astrocytes differentiated from mouse embryonic neural stem cells in the absence of serum. (**a**) Mouse embryonic NSCs were cultured on commonly used plastic plates for 3 days without (control) or with 10 μM Y27632 (Y27632) in serum-free condition. Protein expression of PP-MRLC, total MRLC, and GAPDH (for loading control) was detected by western blotting analysis. The expression levels of (**b**) PP-MRLC and (**c**) total MRLC proteins were normalized relative to GAPDH protein. (**d**) Differentiated cells were assessed by immunofluorescence staining. Neurons were visualized by Tuj1 (green), astrocytes were visualized by GFAP (red), and cell nuclei were counterstained with Hoechst (blue). Scale bar, 100 μm. (**e**) Whole cell number, (**f**) the number of Tuj1 positive cells, and (**g**) the number of GFAP positive cells on each plate were counted and normalized relative to the control. (**h**) Protein expression of Tuj1, GFAP, and GAPDH (for loading control) was detected by western blotting analysis. The expression levels of (**i**) Tuj1 and (**j**) GFAP proteins were normalized relative to GAPDH protein. Error bars represent the standard deviation; *p < 0.05, **p < 0.01, ***p < 0.005 (Student’s *t* test). *ROCK* Rho-associated kinase, *NSCs* neural stem cells, *GAPDH* glyceraldehyde 3-phosphate dehydrogenase, *GFAP* glial fibrillary acidic protein, *PP-MRLC* di-phosphorylated myosin regulatory light chain.

Inhibition of ROCK activity has been reported to reduce the amount of PP-MRLC in various types of cells^24,27–30^. However, to the best of our knowledge, there are no reports that a ROCK inhibitor prevents the di-phosphorylation of MRLC in NSCs. Thus, we examined whether PP-MRLC expression in NSCs was decreased by treatment with Y27632, a common inhibitor of ROCK activity. We cultured mouse embryonic NSCs for 3 days with 10 μM Y27632 on plastic plates in serum-free conditions and examined the levels of PP-MRLC and total MRLC. The results of western blotting showed that the expression of PP-MRLC in NSCs was significantly reduced by treatment with Y27632, whereas total MRLC was not significantly different between untreated and treated cells with Y27632 (Fig. 4a–c). These results showed that treatment with the ROCK inhibitor, Y27632, decreased PP-MRLC in NSCs.

### Differentiation of astrocytes from embryonic NSCs on stiff surfaces is increased by treatment with a ROCK inhibitor in the absence of serum

**Figure 5:**
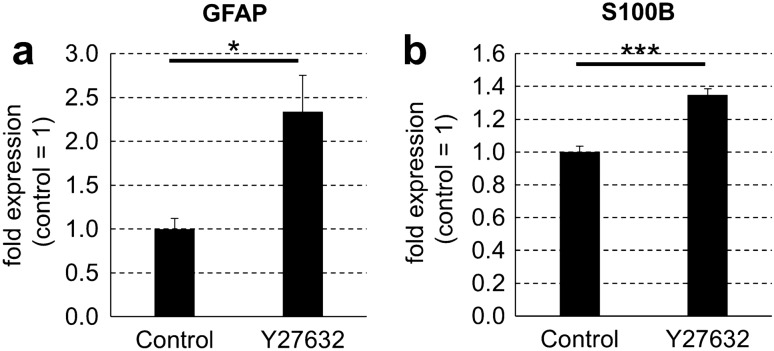
Treatment with ROCK inhibitor increases mRNA expression of astrocyte markers after 3 days of differentiation of mouse embryonic neural stem cells in the absence of serum. (**a**) Mouse embryonic NSCs were cultured on commonly used plastic plates for 3 days without (control) or with 10 μM Y27632 (Y27632) in serum-free condition. mRNA expression of GFAP, S100B and GAPDH on each plate type was detected by qPCR analysis. The expression levels of (**a**) GFAP and (**b**) S100B were normalized relative to that of GAPDH. Error bars represent the standard deviation; *p < 0.05, ***p < 0.005 (Student’s *t* test). *ROCK* Rho-associated kinase, *NSCs* neural stem cells, *GFAP* glial fibrillary acidic protein, *S100B* S100 calcium binding protein B, *GAPDH* glyceraldehyde 3-phosphate dehydrogenase, *qPCR* quantitative polymerase chain reaction.

To determine whether PP-MRLC regulates the differentiation of embryonic NSCs, we treated NSCs with the ROCK inhibitor Y27632 and analyzed the differentiation of the cells into neurons or astrocytes. We cultured mouse embryonic NSCs for 3 days with or without 10 μM Y27632 on plastic plates in serum-free conditions and compared the expression of Tuj1 and GFAP. Immunostaining showed that the whole cell number was not significantly different between the cells; on the other hand, Y27632 treatment significantly decreased the number of Tuj1-positive cells and increased the number of GFAP-positive cells (Fig. 4d–g). Furthermore, western blotting showed that the protein expression of Tuj1 in Y27632-treated NSCs was not significantly different from that in untreated cells (Fig. 4h,i). In contrast, GFAP protein expression in Y27632-treated NSCs was significantly higher than that in untreated cells (Fig. 4h,j and Supplementary Figs. S2-S3). Additionally, qPCR analysis revealed that the mRNA expression of GFAP and S100B was higher in Y27632-treated NSCs than in untreated cells (Fig. 5a,b). Blebbistatin, a myosin II inhibitor, also did not significantly affect the expression of Tuj1 and significantly increased the expression of GFAP in NSCs (Supplementary Fig. S4). Furthermore, calyculin A, which has been reported to increase PP-MRLC^31^, reduced the increase of GFAP expression on soft surfaces (Supplementary Fig. S5). These results suggest that dephosphorylation of MRLC by ROCK inhibition increases the differentiation of embryonic NSCs into astrocytes.

## Discussion

In this study, we found that soft culture surfaces promote the differentiation of embryonic NSCs to astrocytes in serum-free conditions. Contrarily, on stiff culture surfaces, such as the commonly used plastic plates, embryonic NSCs preferably differentiate into neurons in serum-free media. This is the first study to show that astrocytic differentiation of embryonic NSCs is mediated by surface stiffness. Thus far, it is reported that adult NSCs tend to differentiate into neurons on soft culture surfaces, whereas they easily differentiate to astrocytes on stiff culture surfaces regardless of the presence or absence of serum^18–20^. Besides, embryonic NSCs preferably differentiate to neurons in serum-free media on stiff culture surfaces, whereas in a culture medium containing serum, they easily differentiate to astrocytes^21^. Therefore, it is thought that the inconsistency of the differentiation tendency of NSCs induced by stiffness of culture surfaces is due to the difference between adult NSCs and embryonic NSCs, not due to the absence or presence of serum. It is suggested that in soft culture surfaces, adult gliogenic NSCs generate more neurons, whereas embryonic neurogenic NSCs generate more astrocytes. In the present study, we dissected the forebrains of E11.5 mice and created neurospheres for obtaining neurogenic NSCs. To effectively prevent astrocyte differentiation, we differentiated these NSCs within 2 days after dissection (Supplementary Fig. S7). However, the number of NSCs in the neurospheres within 2 days after the dissection was insufficient to perform our experiments. Therefore, we used NSCs proliferated for 4–6 days in neurospheres, while considering the balance between the number of cells required for our experiment and neuronal differentiation. The use of more neurogenic NSCs for the experiments may confirm a much larger effect of soft culture surfaces on astrocytic differentiation.

Differentiation of NSCs is regulated by culturing them in a medium containing serum and/or recombinant proteins, or by passaging them repeatedly^5–8^. Moreover, serum-free conditions are preferable for regenerative medicine because serum might contain unidentified pathogens^9^. In addition, directing the differentiation of NSCs by treating them with recombinant proteins and repeatedly passaging them are costly and time-consuming, respectively. In this study, we showed that the differentiation of embryonic NSCs on culture surfaces of different stiffness in serum-free conditions was confirmed 3 days after seeding. Therefore, we suggest that directing differentiation of embryonic NSCs by culturing them on soft or stiff surfaces with serum-free media is advantageous because this method is quick and does not use an unfavorable serum.

In this report, we used GAPDH as a housekeeping gene to normalize expression. However, changes in the elastic modulus of the surface have been recently proposed to modulate GAPDH activity^32^. Therefore, we also showed that soft culture surfaces or the ROCK inhibitor Y27632 promoted the differentiation of embryonic NSCs into astrocytes in the absence of serum by using beta-actin as a housekeeping protein (Supplementary Figs. S1, S2). Any housekeeping genes/proteins could be affected by surface stiffness; therefore, the use of more than one housekeeping gene for relative quantification is recommended.

Astrocytes are expected to be applicable to regenerative medicine for ALS and SCI because the lifespan of ALS and motor activity of SCI mice models were increased by transplantation of astrocytes to these animals^1–4^. Human iPS cells are expected to be good sources of astrocytes for the treatment of ALS and SCI. However, preparing an adequate amount of astrocytes from iPS cells is time-consuming, especially human cells, because the direct method for differentiating between human astrocytes and human iPS cells are nonexistent, and it takes over 94 days to differentiate human astrocytes from human NSCs owing to their embryonic neurogenic NSC features^12,33,34^. To prepare human astrocytes from human iPS cells, we needed to induce human NSCs from human iPS cells for 12 days by using a dual SMAD (an acronym from the fusion of Caenorhabditis elegans Sma genes and the Drosophila Mad, Mothers against decapentaplegic) inhibition neural differentiation protocol and passage these NSCs for more than 10 times, then differentiate these passaged NSCs into human astrocytes for 14 days in the culture medium containing serum^12^. In the present study, we showed that the differentiation of mouse embryonic NSCs into astrocytes was induced 3 days after seeding on soft surfaces with serum-free media. These results suggest that this rapid method of preparing astrocytes by culturing embryonic NSCs on soft surfaces without serum is better than the conventional methods and may be applicable to human iPS cell-derived NSCs.

Cytokines belonging to the bone morphogenetic protein (BMP) family play important roles in the differentiation of astrocytes from NSCs. It has been reported that activated signal transducer and activator of transcription 3 (STAT3)/Smad1 under the signaling cascade of these cytokines forms a complex with the transcription coactivator p300, and consequently, induces transcriptional activation of astrocyte-specific genes^35–38^. In addition, BMP cascade signaling has been reported to be regulated by the stiffness of culture surfaces^39^. Moreover, previous studies have shown that BMP signaling reduces Nestin expression in NSCs and increases GFAP and S100B expression^8,40^. In this study, we found that the expression of the mature astrocyte marker S100B mRNA was higher in the cells on soft surfaces than on stiff surfaces (Fig. 2a,b). However, qPCR analysis revealed that the mRNA expression of BMP2, BMP4, and BMP8b was lower in the cells on soft surfaces than in the cells on stiff surfaces (Supplementary Fig. S6). Therefore, further studies are required to determine whether BMP cascade signaling is involved in soft-surface-induced astrocyte differentiation. In addition, we believe that differentiation of NSCs on soft surfaces into functional astrocytes progressed because of the upregulation of the mature astrocyte marker S100B mRNA. Conversely, it may be difficult to differentiate NSCs into fully mature astrocytes during the 3-day culture period of this experiment. In addition, it was difficult to examine the effect of the soft culture surface on the functionality of astrocytes accurately because astrocytes proliferated explosively and contacted each other for longer duration (Supplementary Fig. S8). To confirm the functionality of astrocytes differentiated on soft surfaces, it is necessary to culture for longer duration individually or to verify in vivo models of neurodegenerative diseases.

In this study, we used triple coating (poly-D-lysine, laminin, and fibronectin) because this triple coating had the best cell adhesion on culture plates as a result of various examinations on coating. Conversely, previous reports have shown that coating materials affect NSPC characteristics^41^. In addition, differences in the origin of NSCs, the presence or absence of differentiation-inducing factors, and the stiffness of the culture surface were reported to affect astrocytic differentiation^18–20,42,43^. Furthermore, in previous studies examining the effect of stiffness on NSC differentiation, a relatively soft range (1–30 kPa) was used in some studies, whereas a hard range (10–10,000 kPa) was used in some other studies^18,19^. In this study, we examined the effect of stiffness on NSC differentiation within a relatively wide range (1 kPa–2.8 GPa). Since differences in the effect of stiffness on NSC differentiation have been reported even in a relatively narrow range, different results may be obtained if we examine a narrower range of stiffness in our experimental system. Therefore, further studies are required to determine the optimal differentiation method for astrocytes.

NSCs differentiate into astrocytes on their own through cell division^44^. In this study, Y27632 promoted the differentiation of NSCs into astrocytes such that cell division of NSCs was reduced and the total number of Y27632 treated cells was expected to decrease. In contrast, the results showed that there was no change in the number of cells after 3 days of culture (Fig. 4e). These results suggest that the cell survival effect of Y27632 contributed to the maintenance of the cell number during differentiation, though the cell division of NSCs was reduced.

The results of this study showed that PP-MRLC of NSCs was decreased by culturing on soft surfaces or by treating them with inhibitors, the ROCK inhibitor Y27632, and the myosin II inhibitor blebbistatin. A previous study showed that treatment with blebbistatin prevented surface stiffness-dependent differentiation of brain, muscle, and bone cells from mesenchymal stem cells^13^. Furthermore, stiff surfaces induced osteogenic differentiation of mesenchymal stem cells dependent on RhoA activity, which is critical for the contraction of actomyosin^45^. Therefore, it is possible that surface stiffness regulates the differentiation of various types of stem cells, including NSCs and mesenchymal stem cells, by modulating cellular contractile forces generated by actomyosin. In addition, some previous reports have shown the additive effects of soft substrate and Y27632 in various cells other than NSCs^24,46,47^. The use of Y27632 in addition to a soft culture surface may have a substantial effect on astrocytic differentiation from NSCs.

The results of the present study showed that astrocytic differentiation of embryonic NSCs was induced by culturing them on soft surfaces in the absence of serum. In addition, we also found that astrocytic differentiation of NSCs triggered by soft surfaces was dependent on the dephosphorylation of MRLC. These results suggest that culturing embryonic NSCs on soft surfaces or treating them with the drug to reduce MRLC activity might be a suitable method to prepare astrocytes in regenerative medicine.

## Methods

### Preparing culture plates

In this study, the commonly used polystyrene culture plates (3815–012; Iwaki, Tokyo, Japan, or 3513; Corning, NY, USA) and Softwell culture plates (Matrigen, CA, USA) with stiffness of 1 kPa (SW12-EC-1 EA) and 12 kPa (SW12-EC-12 EA) for culturing cells on surfaces with various stiffnesses were utilized. The stiffness of the polystyrene culture plate and Softwell at 1 kPa and 12 kPa under the conditions used in this study were measured using atomic force microscopy (Nano Wizard 4; JPK, Berlin, Germany). The results indicated that the stiffness of the polystyrene culture plate was 2.8 GPa (SD: 0.85 GPa), and that of plates with 1 kPa and 12 kPa was 0.560 kPa (SD: 0.146 kPa) and 15.697 kPa (SD: 2.390 kPa), respectively. Culture plates were coated with poly-d-lysine, laminin, and fibronectin before seeding the cells. To coat these molecules, aliquots of 0.5 mL of 20 μg/mL poly-d-lysine (Corning) in sterile phosphate-buffered saline (PBS) were placed in the wells of the culture plates. The plates were then incubated overnight at 37 °C. The next day, the plates were rinsed twice with sterile PBS and incubated with 0.5 mL of 10 μg/mL mouse laminin (Thermo Fisher Scientific, MA, USA) and 0.5 μg/mL human fibronectin (Thermo Fisher Scientific) in sterile PBS for at least 3 h at 37 °C. The plates were rinsed twice with sterile PBS prior to use.

### Isolating and culturing embryonic mouse NSCs

Embryonic mouse NSCs were obtained via the modification of a previously described method^48^. Briefly, pregnant female C57BL/6JJcl mice were purchased from CLEA Japan and dissected after cervical spine dislocation with isoflurane anesthesia (871,129; Pfizer, NY, USA). E11.5 mice were taken out from these mice and the forebrains of the E11.5 mice were dissected and mechanically dispersed. Aliquots of 50,000 cells/mL mouse NSCs in KBM neural stem cell medium (KOHJIN BIO, Saitama, Japan) were cultured in ultra-low attachment culture flasks (3814; Corning). The culture medium was added twice daily. The cells were passaged into new flasks 3 days after seeding. Four to six days after seeding, the neurospheres that had formed were rinsed in sterile PBS and dispersed with Accutase (Innovative Cell Technologies, CA, USA) at 37 °C for 5 min. The dispersed NSCs were used for subsequent experiments. Total number of pregnant female C57BL/6JJcl mice in this study was 36 for obtaining adequate number of NSCs needed for our experiments. Animal care and experimental procedures were approved by Shionogi’s Institutional Animal Care and Use Committee. Animal care and experimental procedures were performed in accordance with guidelines provided by Shionogi’s Institutional Animal Care and Use Committee. The study was carried out in accordance with the ARRIVE guidelines (https://arriveguidelines.org).

### Differentiation of NSCs

After washing with sterile PBS, the NSCs were dissociated into single cells with Accutase. Before seeding the cells on poly-d-lysine, laminin, and fibronectin-coated 12-well plates, the wells were filled with 500 μL of Neurobasal plus B27 medium (Thermo Fisher Scientific) with 0.5% Dimethyl sulfoxide (DMSO), 500 μL of 20 μM Y27632 (Wako, Osaka, Japan) in Neurobasal plus B27 medium with 0.5% DMSO, or 50 μM blebbistatin (Sigma Aldrich, MO, USA) in Neurobasal plus B27 medium with 0.5% DMSO, or 0.2 nM Calyculin A (Wako) in Neurobasal plus B27 medium with 0.5% DMSO. Then, 500,000 cells in 500 μL of Neurobasal plus B27 medium were added to each well. The medium was changed to 500 μL of Neurobasal plus B27 medium with 0.25% DMSO, 500 μL of 10 μM Y27632 in Neurobasal plus B27 medium with 0.25% DMSO, or 500 μL of 25 μM blebbistatin in Neurobasal plus B27 medium with 0.25% DMSO, or 500 μL of 0.1 nM Calyculin A in Neurobasal plus B27 medium with 0.5% DMSO of daily. After 3 days of culture, the cells were analyzed.

### Immunocytochemical staining

Cells were fixed with 4% paraformaldehyde in PBS for 15 min at room temperature. The cells were permeabilized with 0.2% Triton-X100 in PBS with 1% normal goat serum (Vector Laboratories, CA, USA) for 60 min at room temperature and incubated with primary antibodies in PBS containing 1% normal goat serum at 4 °C overnight. After washing the primary antibodies with PBS, the cells were incubated with secondary antibodies conjugated with Alexa Fluor® 488 anti-mouse IgG (1:500; Invitrogen, CA, USA) in PBS or Alexa Fluor® 555 anti-rabbit IgG (1:500; Invitrogen) in PBS for 60 min at room temperature. The nuclei were stained with Hoechst 33423 (1:10,000; Invitrogen) for 15 min at room temperature. The commercial antibodies used for immunostaining were as follows: Dako for GFAP (rabbit polyclonal/M0761/1:2,000) and Covance for Tuj1 (mouse monoclonal/MMS-435P/1:500). Stained cells were observed and imaged using a fluorescent microscope with a 20 × objective (BZ-9000; Keyence, Osaka, Japan). For the quantification of immunostaining, four fields per well were analyzed, and the average number of cells was calculated for three wells in three independent experiments (animal preparation). To calculate the population of astrocytes or neurons, strong Hoechst 33423-positive cells with smaller nuclei (dead cells) were excluded from the analysis. Then, we counted the number of cells with Hoechst 33423 positive nuclei inside the GFAP-positive area or Tuj1 positive area.

### Western blotting

For the detection of GFAP, Tuj1, glyceraldehyde 3-phosphate dehydrogenase (GAPDH) and beta actin, the cells were lysed in radioimmunoprecipitation assay buffer (RIPA buffer, Sigma Aldrich) with protease inhibitors (Roche Life Science, Penzberg, Germany) and incubated for 3 min at 95 °C with the addition of sample buffer solution (Nacalai Tesque, Kyoto, Japan). Proteins in the cell lysates were separated by sodium dodecyl sulfate (SDS)-polyacrylamide gel electrophoresis with 5–20% polyacrylamide gels (e-PAGEL; ATTO, Tokyo, Japan) using Tris–glycine-SDS running buffer (Thermo Fisher Scientific) and transferred onto polyvinylidene fluoride membranes (iBlot gel transfer PVDF; Invitrogen). The membranes were blocked with 5% skim milk (Nacalai Tesque) in Tris-buffered saline-Tween 20 (TBS-T, Cell Signaling, MA, USA) for 60 min at room temperature and incubated with 1:1,000 anti-GAPDH (14C10) rabbit monoclonal antibody (Cell Signaling) in 1% bovine serum albumin (BSA)/TBS-T or 1:1,000 anti-beta actin (13E5) rabbit monoclonal antibody (Cell Signaling) in 1% bovine serum albumin (BSA)/TBS-T at 4 °C overnight. After washing with TBS-T, the membranes were incubated with 1:2000 anti-rabbit IgG horseradish peroxidase (HRP)-linked antibody (Cell Signaling) in 1% BSA/TBS-T for 60 min at room temperature. Signal emission was induced using ECL Prime Western Blotting Detection Reagent (GE Healthcare, IL, USA). Imaging of ECL blots and densitometric analysis was performed using LAS 3000 luminoimage analyzer (FUJIFILM, Tokyo, Japan). After detection, antibodies on the membrane were detached by incubation with stripping solution (Wako) for 120 min at 37 °C and incubated with 1:2,000 anti-GFAP (D1F4Q) rabbit monoclonal antibody (Cell Signaling) in 1% BSA/TBS-T or anti-Tuj1 (D71G9) rabbit monoclonal antibody (Cell Signaling) in 1% BSA/TBS-T at 4 °C overnight. After washing with TBS-T, the membranes were incubated with 1:2,000 anti-rabbit IgG HRP-linked antibody in 1% BSA/TBS-T for 60 min at room temperature. Signal emission was detected in the same manner as described above.

For the detection of PP-MRLC and total MRLC, the cells were fixed in cold 10% trichloroacetic acid (Sigma Aldrich) in PBS for 5 min on ice. The cells were washed three times with cold PBS for 3 min on ice and then lysed in SDS buffer (0.125 M Tris–HCl, 0.2 M dithiothreitol, 4% SDS, 20% glycerol, and 0.01% bromophenol blue, pH 6.8). The cell lysates were subjected to ultrasonic fragmentation and incubated at 95 °C for 5 min. Proteins in the cell lysates were separated using 10% SDS–polyacrylamide gels and then transferred onto polyvinylidene fluoride membranes. The membranes were prepared separately for total MRLC and PP-MRLC detection because of the same molecular weight (18 kDa) between them. The membranes were then incubated with the appropriate primary antibody (rabbit monoclonal/3674/1:1,000 for PP-MRLC, or rabbit monoclonal/3672/1:1,000 for total MRLC; Cell Signaling) in Can Get Signal Solution A at 4 °C overnight. Membranes were then washed three times with TBS-T (10 mM Tris–HCl containing 150 mM NaCl and 0.05% Tween 20, pH 7.5) and incubated with the appropriate secondary antibody (HRP anti-rabbit IgG: 1:10,000 for PP-MRLC, HRP anti-rabbit IgG: 1:5,000 for total MRLC; Cell Signaling) in Can Get Signal Solution B for 1 h at room temperature. Protein signals were detected using an Immobilon Western Chemiluminescent HRP substrate (EMD Millipore, MA, USA). We measured MRLC with GAPDH or PP-MRLC with GAPDH on the same membrane, and GAPDH-normalized data were used in the study.

For the quantification of western blots, we seeded 500,000 cells/well in 12 well plates (131,579 cells/cm^2^) and extracted protein from three wells and collected them into one after 3 days of culture to analyze the expression level of protein in an experiment and calculated the average expression level with GAPDH normalized in three independent experiments (animal preparation). The uncropped blots are shown in Supplementary Fig. S9.

### Real-time quantitative polymerase chain reaction (qPCR)

Total ribonucleic acid (RNA) was prepared from cells cultured under various conditions using TRIzol reagent (Life Technologies) according to the manufacturer’s instructions. Reverse transcription was carried out using 1 μg of total RNA in a final volume of 1000 μL using SuperScript III First-Strand Synthesis SuperMix (Life Technologies) according to the manufacturer’s instructions. Real-time qPCR was performed with the 7500 Real-Time PCR System using Power SYBR Green PCR Master Mix (Applied Biosystems, Foster City, CA, USA). The following primers were used and purchased from Takara Bio Inc. (Shiga, Japan): GFAP (forward: 5'-GACCAGCTTACGGCCAACAG-3', reverse: 5'-TCTATACGCAGCCAGGTTGTTCTC-3'), S100B (forward: 5'-ATCAACAACGAGCTCTCTCACTTCC-3', reverse: 5'-TCGTCCAGCGTCTCCATCAC-3'), Nestin (forward: 5'-GAGGTGTCAAGGTCCAGGATGTC-3', reverse: 5'-GACACCGTCTCTAGGGCAGTTACAA-3’), and GAPDH (forward: 5'-TGTGTCCGTCGTGGATCTGA-3', reverse: 5'-TTGCTGTTGAAGTCGCAGGAG-3').

## Supplementary Information

Supplementary Information.

## Data Availability

All data generated or analyzed during this study are included in this published article and the Supplementary Information file.
