# Supplementary material for: Soft surfaces promote astrocytic differentiation of mouse embryonic neural stem cells via dephosphorylation of MRLC in the absence of serum
Source: Sci Rep. 2021 Oct 1;11:19574. doi: 10.1038/s41598-021-99059-5 (PMC8486742; doi:10.1038/s41598-021-99059-5)

Soft surfaces promote astrocytic differentiation of mouse embryonic neural stem cells via dephosphorylation of MRLC in the absence of serum

Hiroshi Oyama, Akihiro Nukuda, Seiichiro Ishihara, Hisashi Haga

Submission ID: 2bfbb731-0fdd-46dc-8e7c-cb23f3eb30ed

Supplementary Figure S1

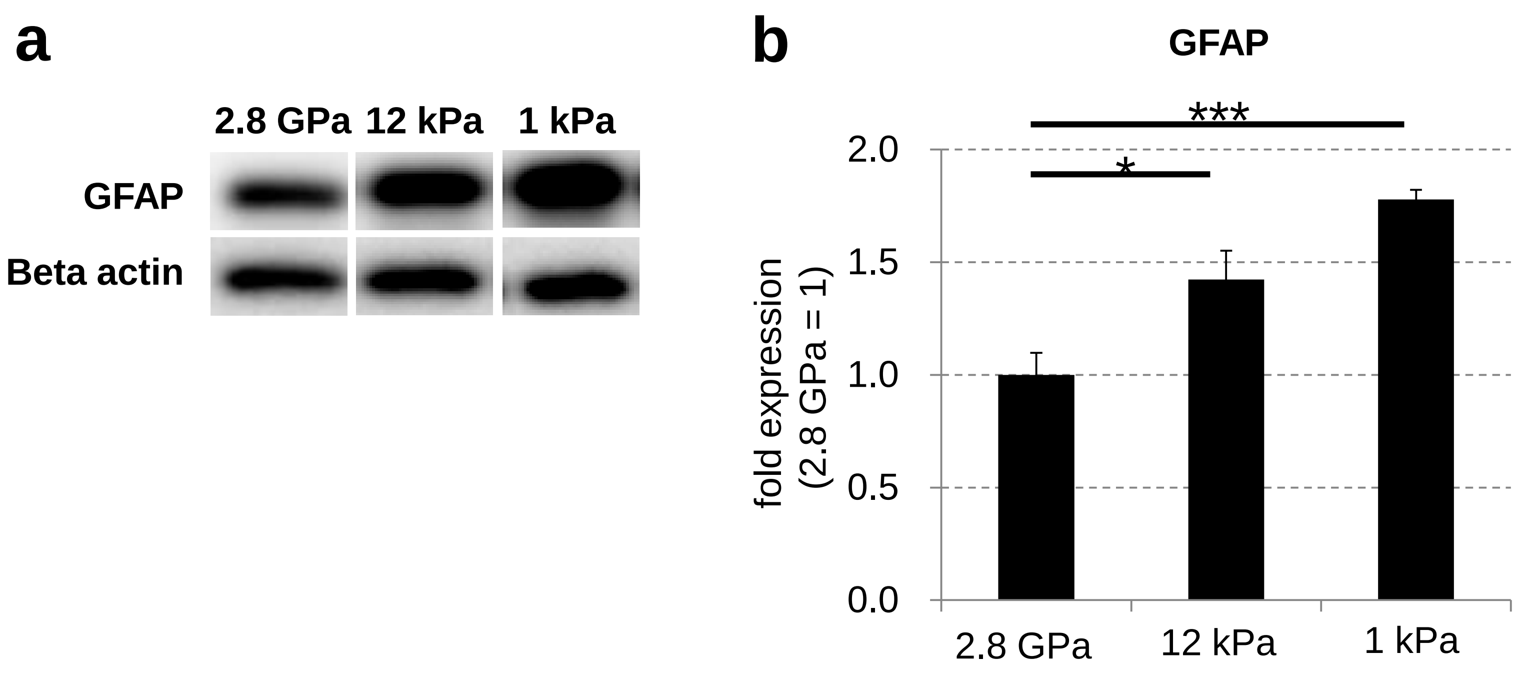

Fig. S1. Soft surfaces increase GFAP expression after 3 days of differentiation of mouse embryonic neural stem cells in the absence of serum

(a) Mouse embryonic NSCs were cultured on three types of plates (1 kPa plates, 12 kPa plates and commonly used plastic plates [2.8 GPa]) for 3 days in serum-free condition. Protein expression of GFAP, and beta actin (for loading control) on each plate was detected by western blotting analysis. The expression levels of (b) GFAP proteins were normalized relative to beta actin protein. Error bars represent the standard deviation; *$p < 0.05$, ***$p < 0.005$ (Student's *t* test with Bonferroni correction).

Abbreviations: NSCs: neural stem cells; GFAP: glial fibrillary acidic protein

Supplementary Figure S2

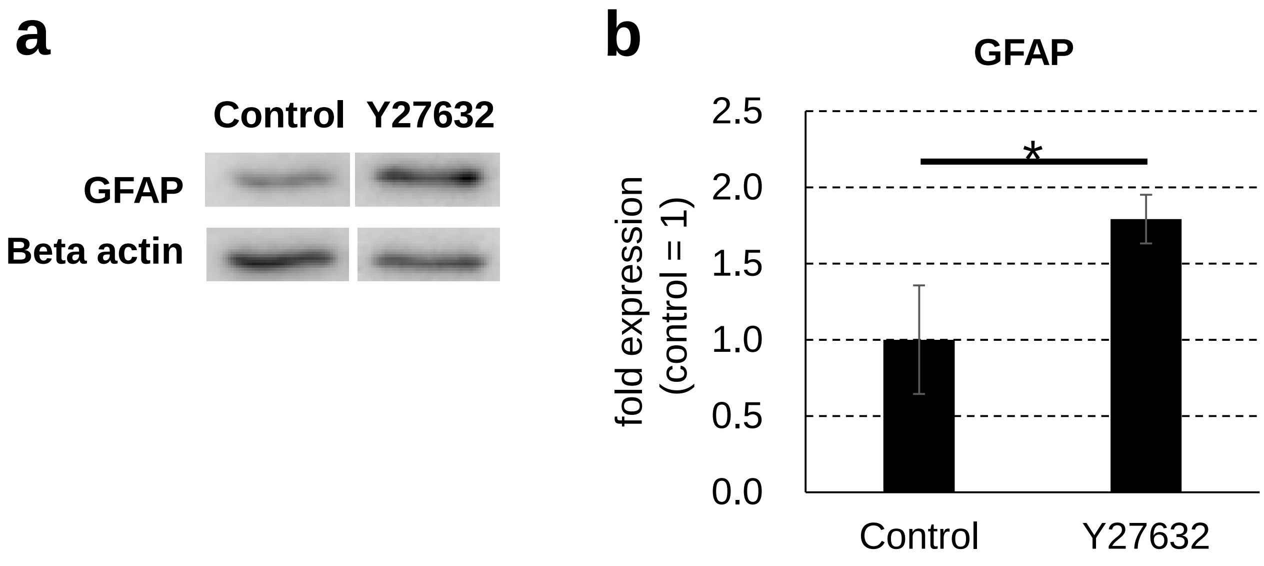

Fig. S2. ROCK inhibitor increases GFAP expression of differentiated cells from mouse embryonic neural stem cells in the absence of serum

(a) Mouse embryonic NSCs were cultured on commonly used plastic plates for 3 days without (control) or with 10 μM Y27632 (Y27632) in serum-free condition. Protein expression of GFAP, and beta actin (for loading control) was detected by western blotting analysis. The expression levels of (b) GFAP proteins were normalized relative to beta actin protein. Error bars represent the standard deviation; *$p < 0.05$ (Student's *t* test).

Abbreviations: ROCK, Rho-associated kinase; NSCs, neural stem cells; GFAP, glial fibrillary acidic protein

Supplementary Figure S3

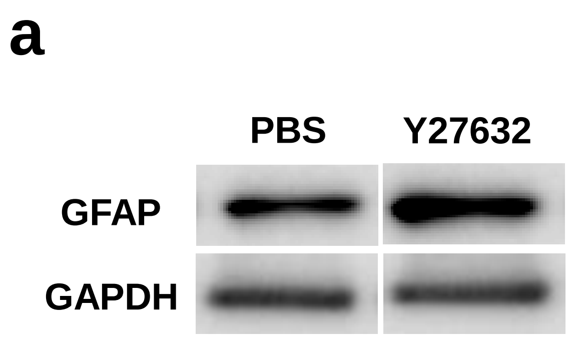

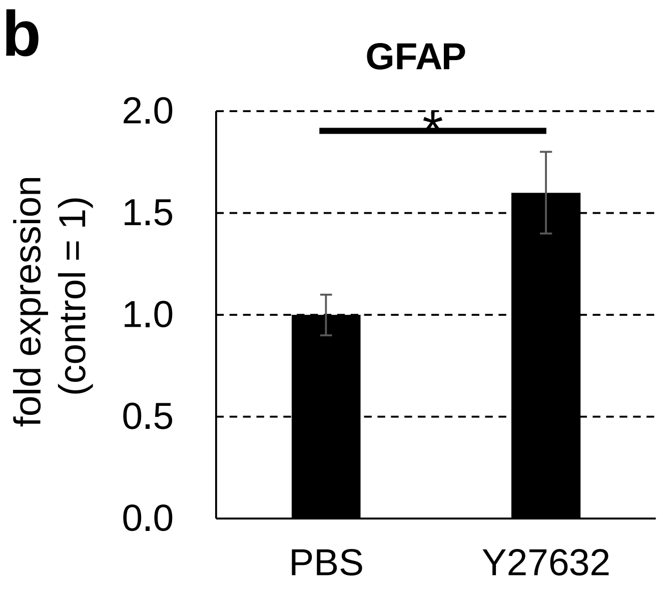

Fig. S3. ROCK inhibitor increases GFAP expression of differentiated cells from mouse embryonic neural stem cells in the absence of serum
(a) Mouse embryonic NSCs were cultured on commonly used plastic plates for 3 days without (PBS) or with 10 μM Y27632 (Y27632) in serum-free condition. Protein expression of GFAP, and GAPDH (for loading control) was detected by western blotting analysis. The expression levels of (b) GFAP proteins were normalized relative to GAPDH protein. Error bars represent the standard deviation; *$p < 0.05$ (Student's *t* test).
Abbreviations: PBS, phosphate-buffered saline; ROCK, Rho-associated kinase; NSCs, neural stem cells; GFAP, glial fibrillary acidic protein; GAPDH, glyceraldehyde 3-phosphate dehydrogenase.

Supplementary Figure S4

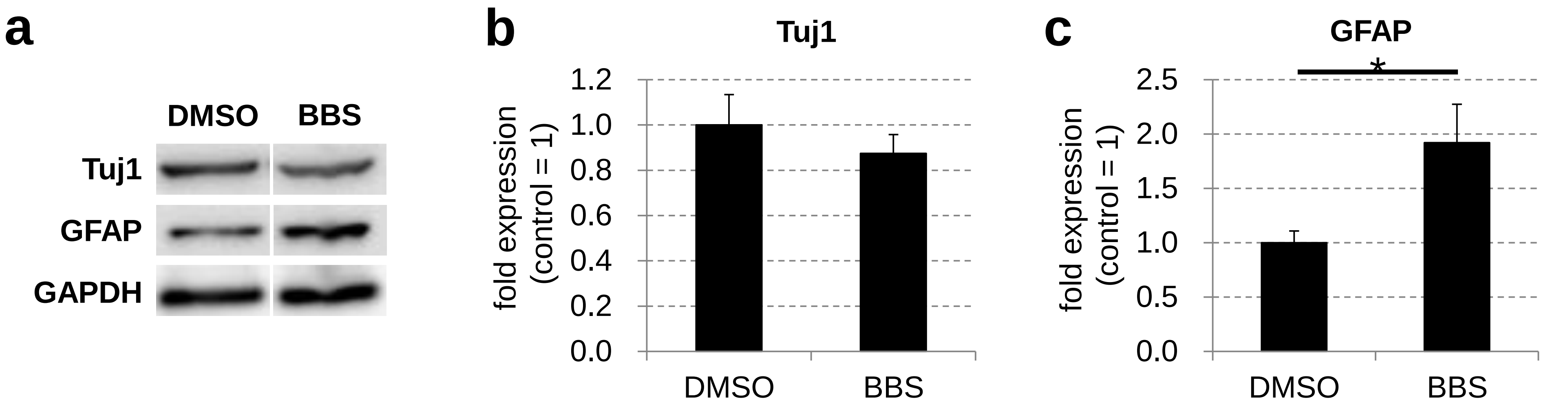

Fig. S4. Blebbistatin increases the GFAP expression of differentiated cells from mouse embryonic neural stem cells in the absence of serum

(a) Mouse embryonic NSCs were cultured on commonly used plastic plates for 3 days without (DMSO) or with 25 μM blebbistatin (BBS) in serum-free condition. Protein expression levels of Tuj1, GFAP, and GAPDH (for loading control) was detected by western blotting analysis. The expression levels of (b) Tuj1 and (C) GFAP proteins were normalized relative to GAPDH protein. Error bars represent the standard deviation; *$p < 0.05$ (Student's *t* test).

Abbreviations: NSCs: neural stem cells; DMSO: Dimethyl sulfoxide; GAPDH: glyceraldehyde 3-phosphate dehydrogenase; GFAP: glial fibrillary acidic protein; PP-MRLC: di-phosphorylated myosin regulatory light chain

Supplementary Figure S5

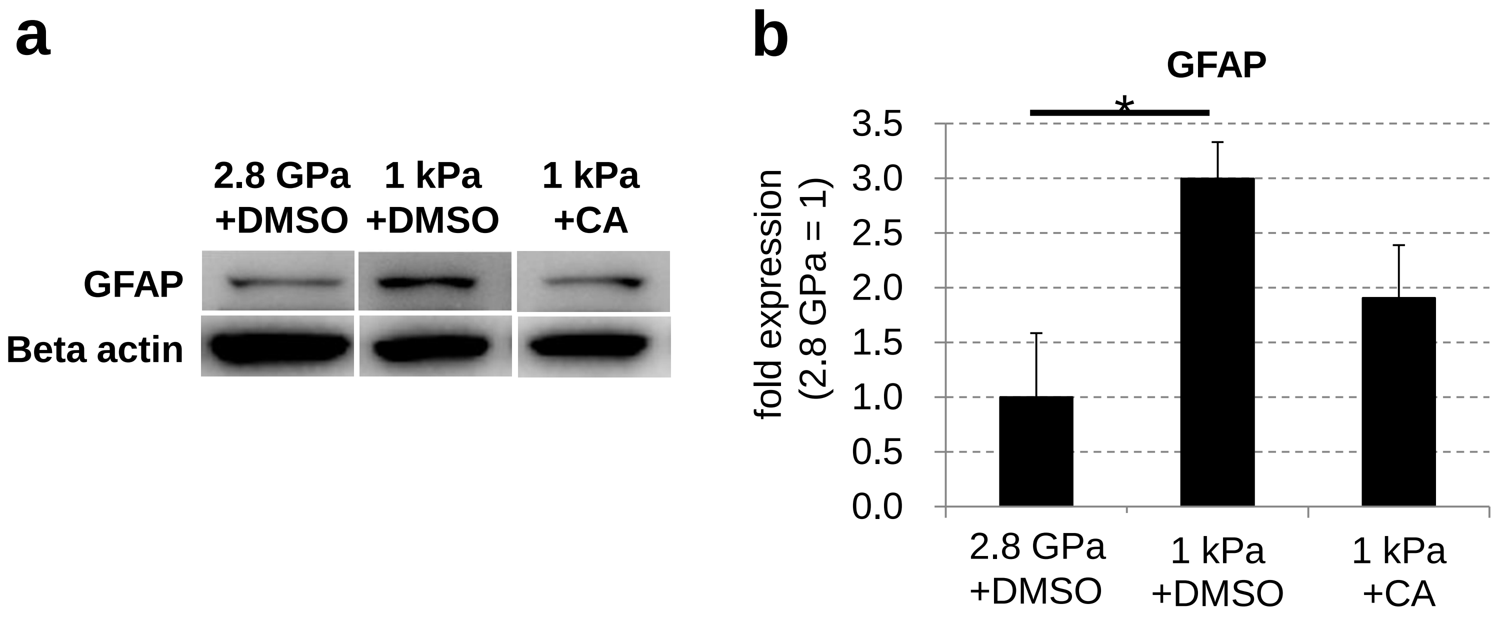

Fig. S5. Calyculin A inhibits the increase of GFAP expression of differentiated cells from mouse embryonic neural stem cells on a soft surface in the absence of serum

(a) Mouse embryonic NSCs were cultured on two types of plates (1 kPa plates and commonly used plastic plates [2.8 GPa]) for 3 days without (DMSO) or with 0.1 nM Calyculin A (CA) in serum-free condition. Protein expression of GFAP, and beta actin (for loading control) was detected by western blotting analysis. The expression levels of (b) GFAP proteins were normalized relative to beta actin protein. Error bars represent the standard deviation; *$p < 0.05$ (Student's t test with Bonferroni correction).

Abbreviations: NSCs: neural stem cells; DMSO: dimethyl sulfoxide; GFAP: glial fibrillary acidic protein.

Supplementary Figure S6

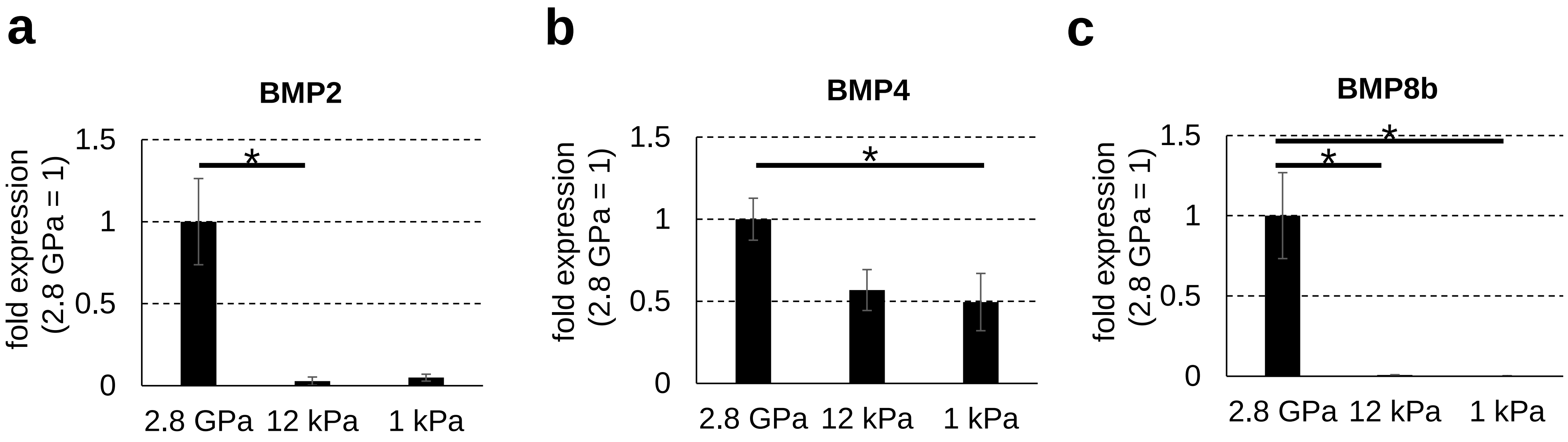

Fig. S6. Soft surfaces decrease expression of BMPs after 3 days of differentiation of mouse embryonic neural stem cells in the absence of serum

(a) Mouse embryonic NSCs were cultured on three types of plates (1 kPa plates, 12 kPa plates, and the commonly used plastic plates [2.8 GPa]) for 3 days under serum-free condition. The mRNA expression of BMP2, BMP4, BMP8b, and GAPDH on each plate type was detected using qPCR analysis. The expression levels of (a) BMP2, (b)BMP4, and (c)BMP8b were normalized relative to GAPDH. Error bars represent the standard deviation; *$p < 0.05$ (Student's *t* test with Bonferroni correction).

Abbreviations: NSCs: neural stem cells; BMP, bone morphogenetic protein; GAPDH, glyceraldehyde 3-phosphate dehydrogenase; qPCR, quantitative polymerase chain reaction.

Supplementary Figure S7

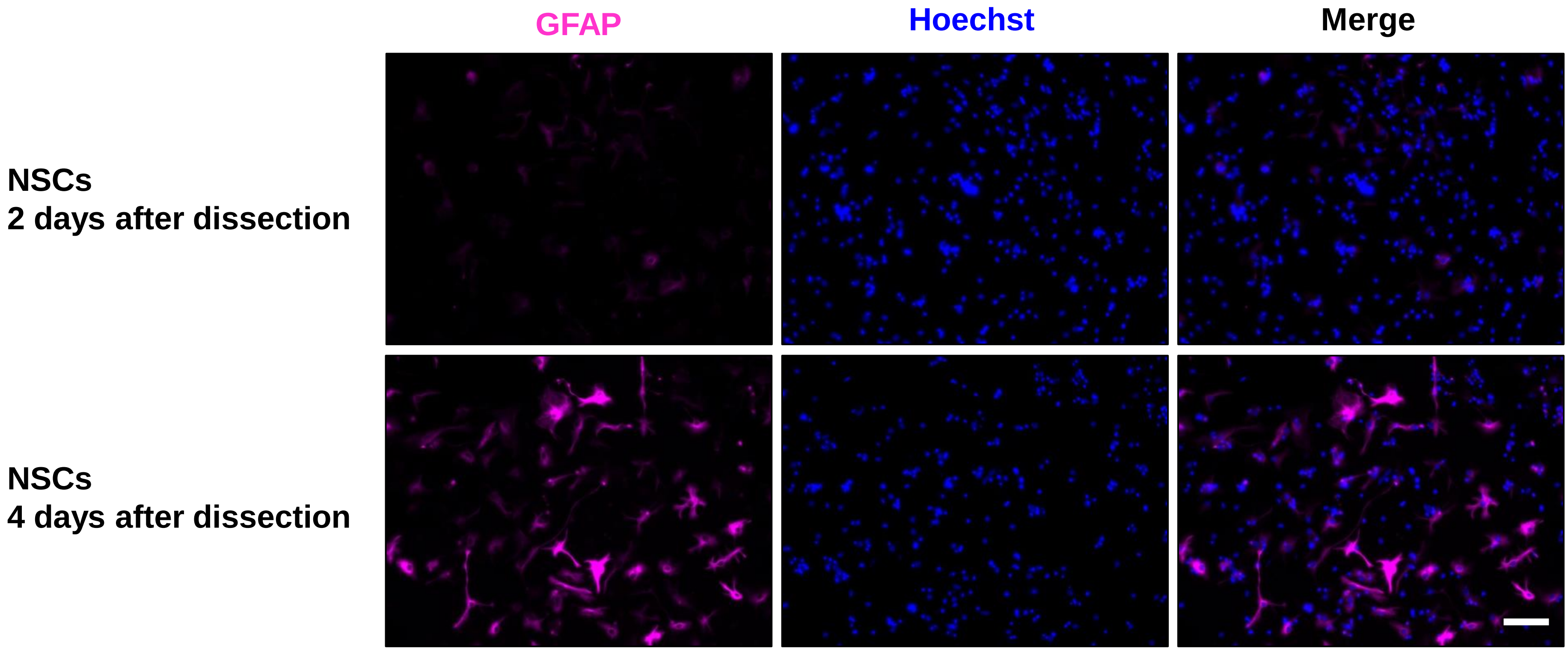

Fig. S7. Culture days after dissections increase astrocytes differentiation from mouse embryonic neural stem cells

Mouse embryonic NSCs 2 days after dissection or NSCs 4 days after dissection were cultured on commonly used plastic plates [2.8 GPa] for 3 days under serum-free conditions and assessed by immunofluorescence staining. Astrocytes were visualized using GFAP (purple), and the cell nuclei were counterstained with Hoechst (blue). Scale bar, 100 μm.

Abbreviations: NSCs: neural stem cells; GFAP: glial fibrillary acidic protein.

Supplementary Figure S8

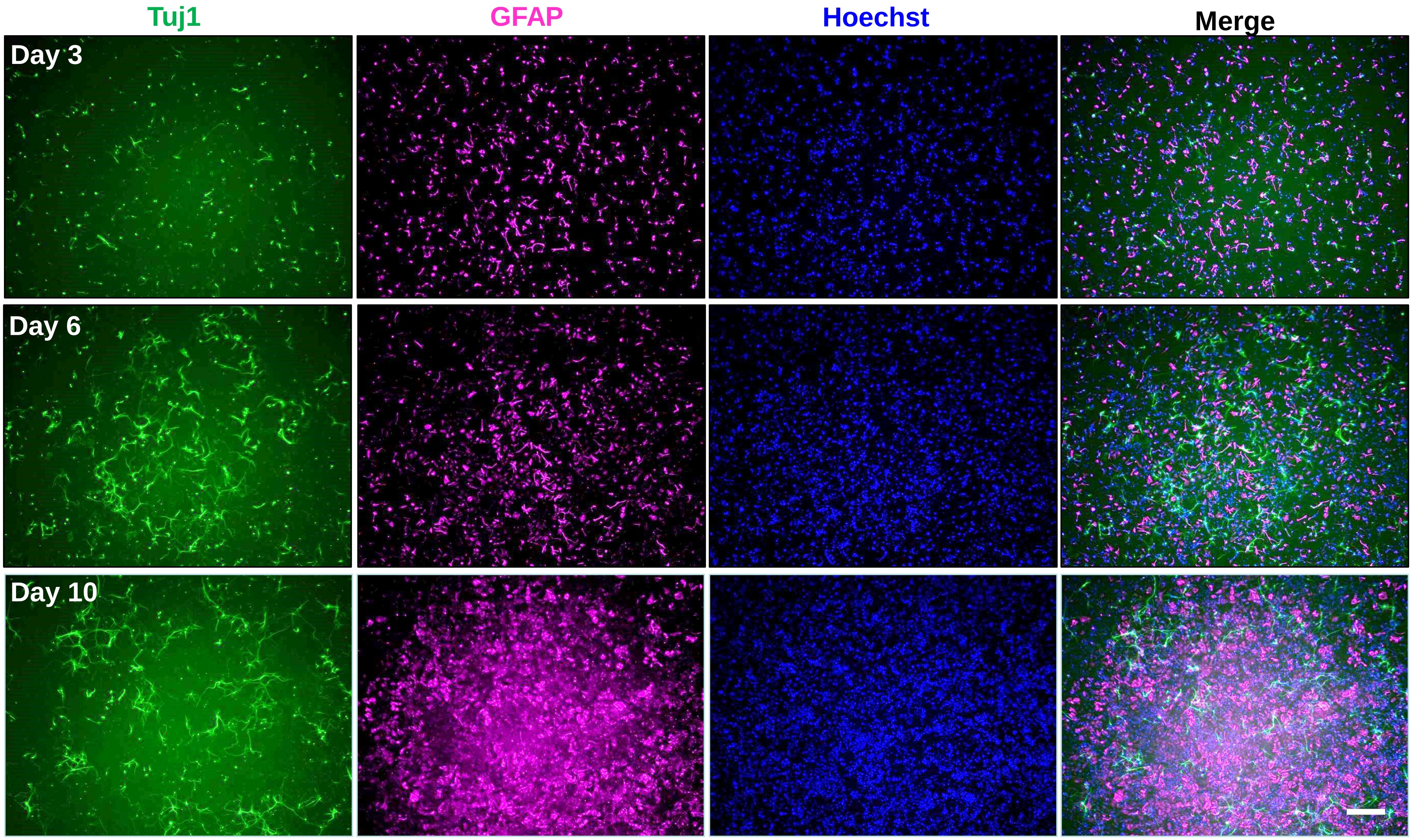

Fig. S8. Increase in astrocytes following culture for long duration
Mouse embryonic NSCs were cultured on 1 kPa plates for 3, 6, and 10 days in serum-free conditions and assessed by immunofluorescence staining. Neurons were visualized with Tuj1(green), astrocytes were visualized using GFAP (purple), and cell nuclei were counterstained with Hoechst (blue). Scale bar, 200 μm.
Abbreviations: NSCs: neural stem cells; GFAP: glial fibrillary acidic protein.

Supplementary Figure S9

Extended data figure 1e

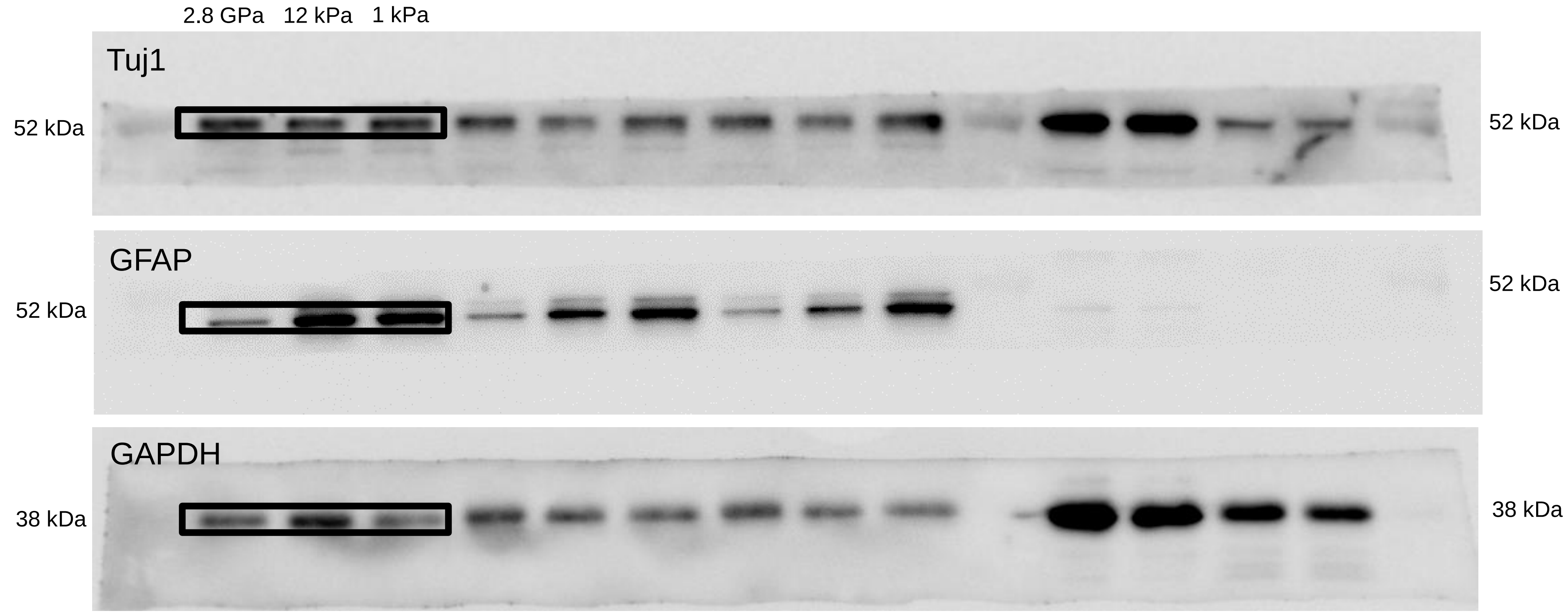

Extended data figure 3a

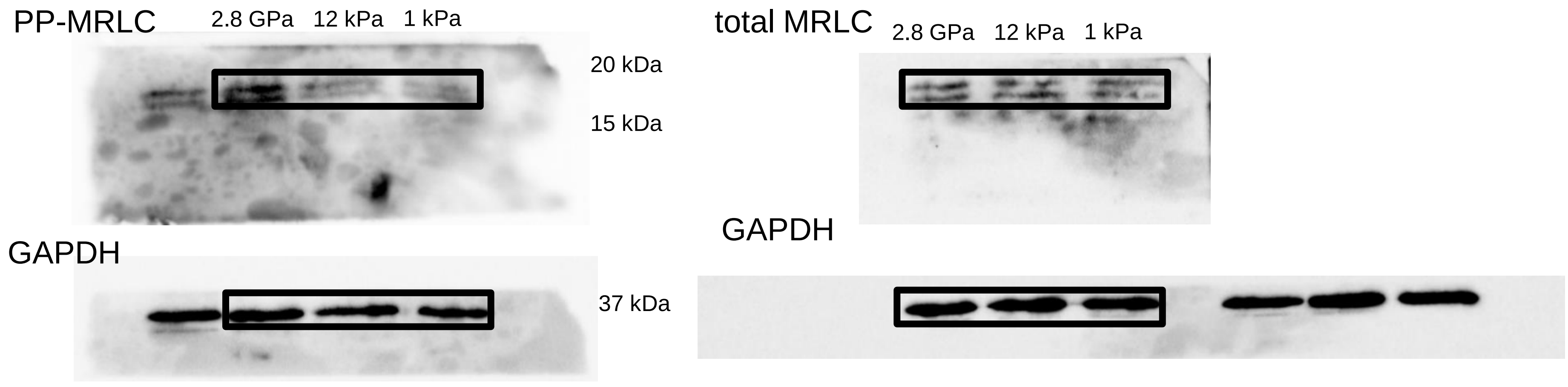

Extended data figure 4a

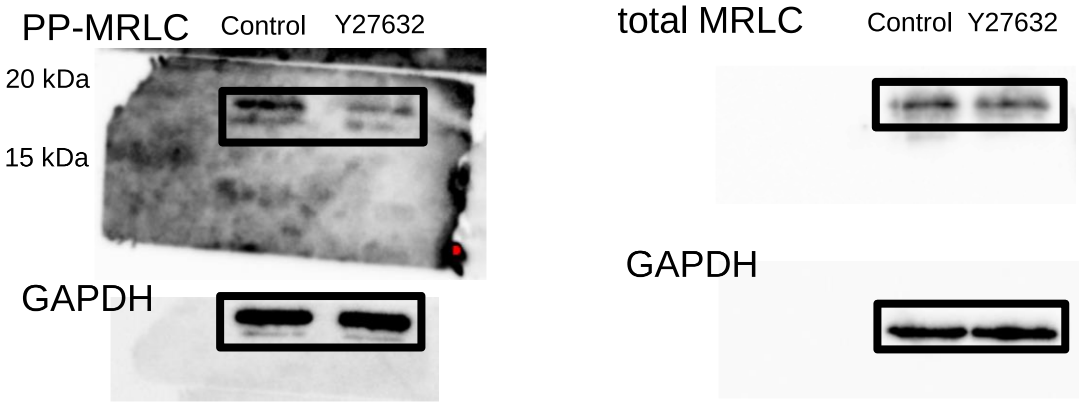

Extended data figure 4h

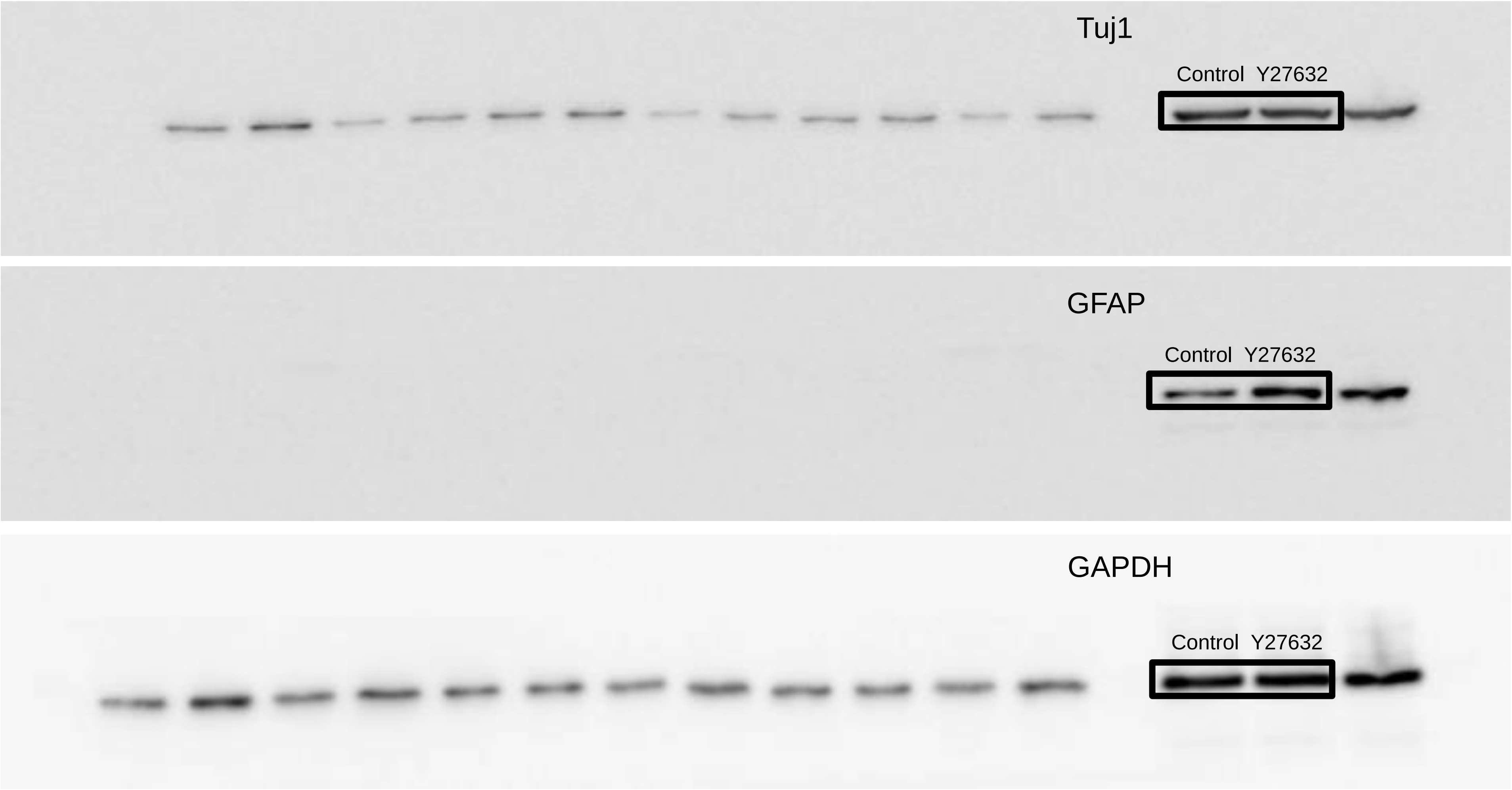

Extended data supplementary figure S1a

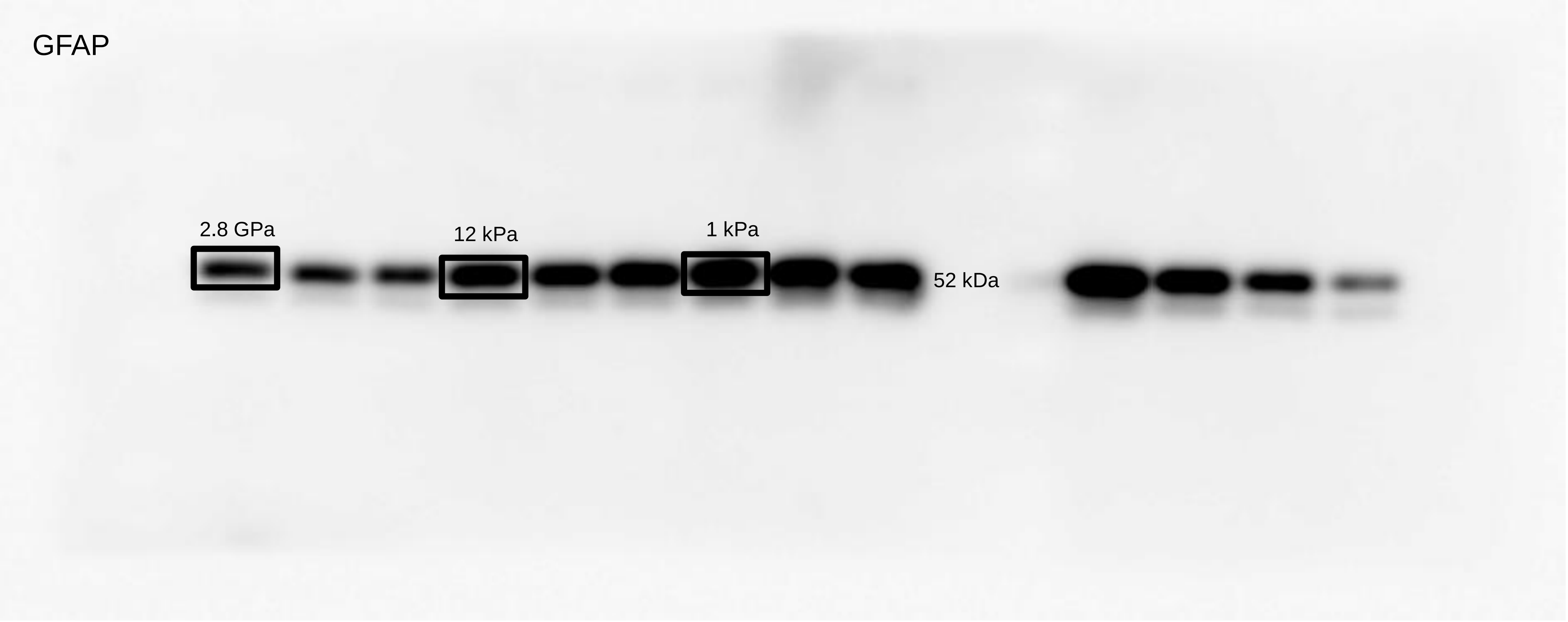

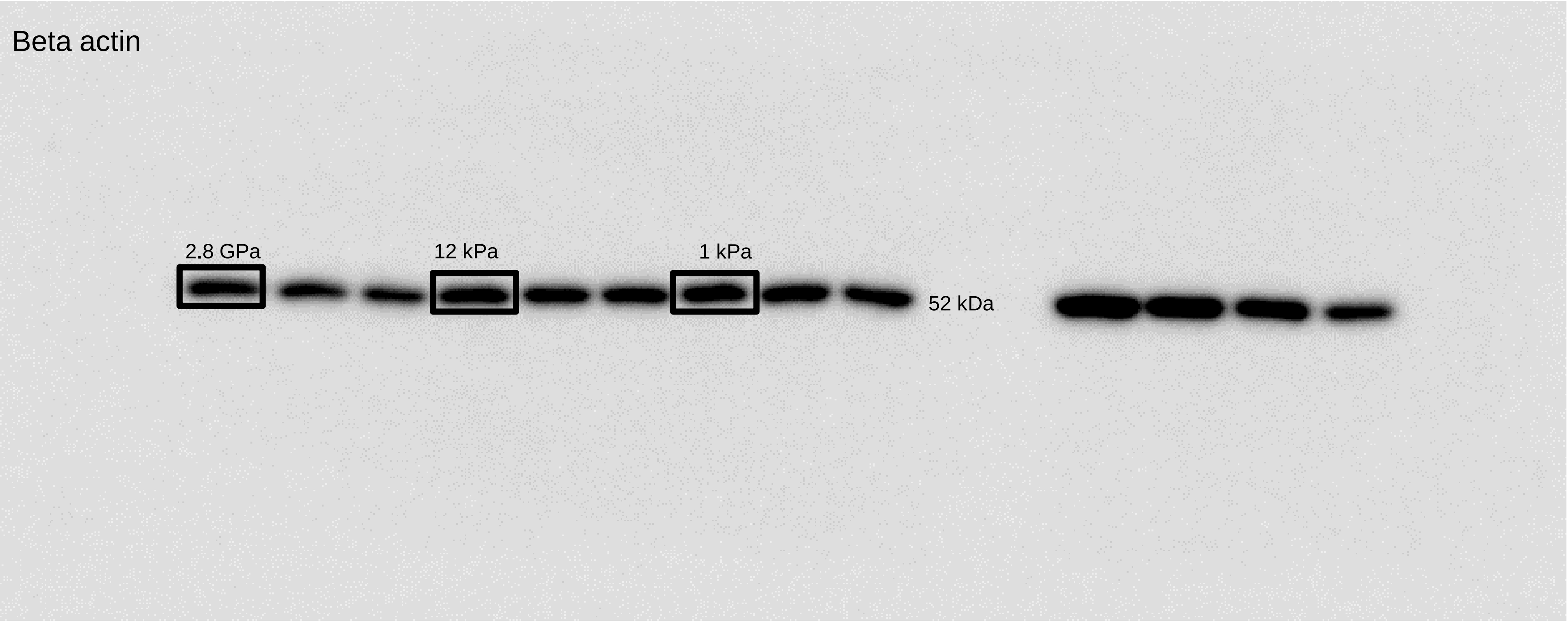

Extended data supplementary figure S2a

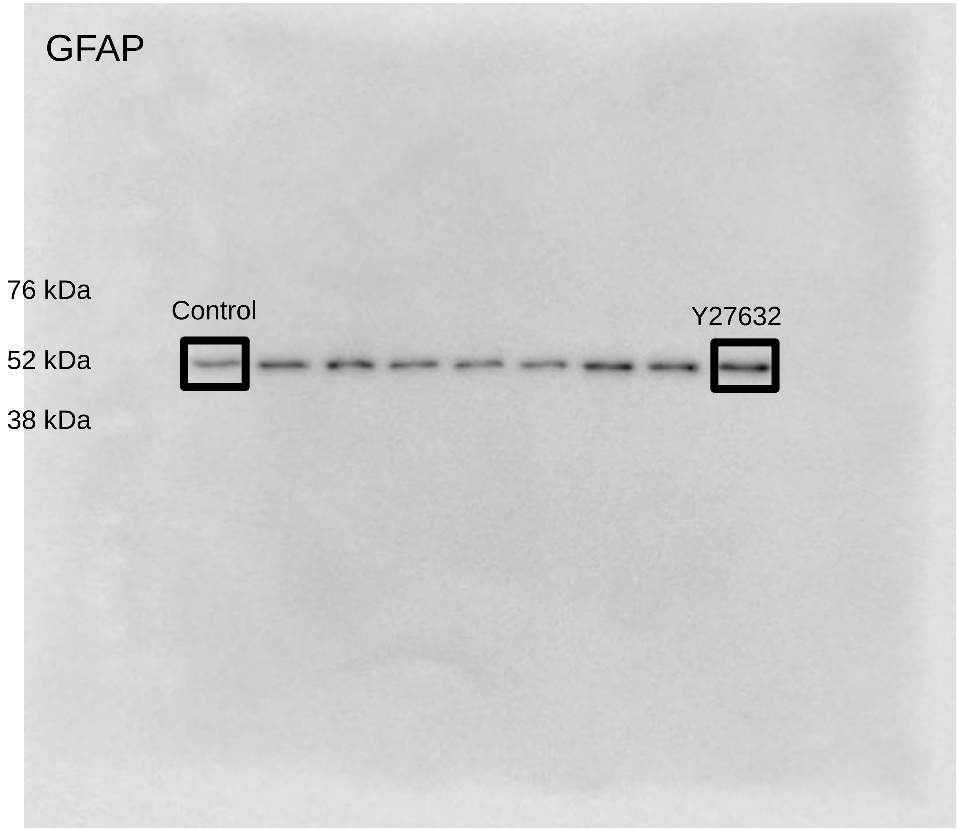

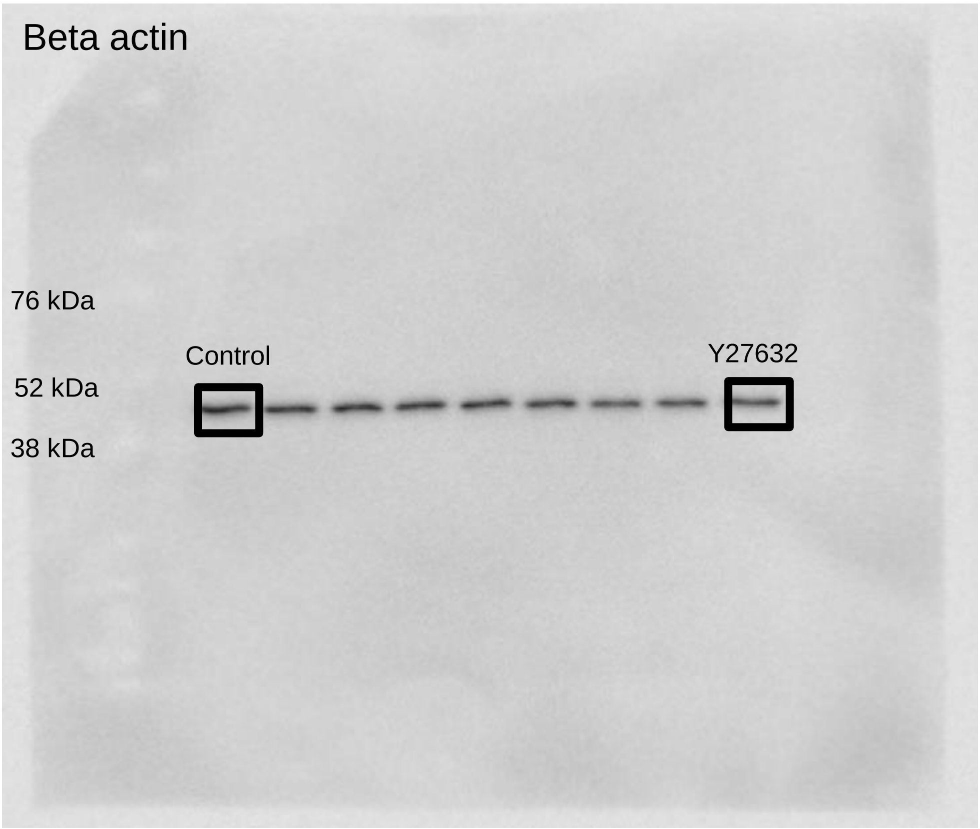

Extended data supplementary figure S3a

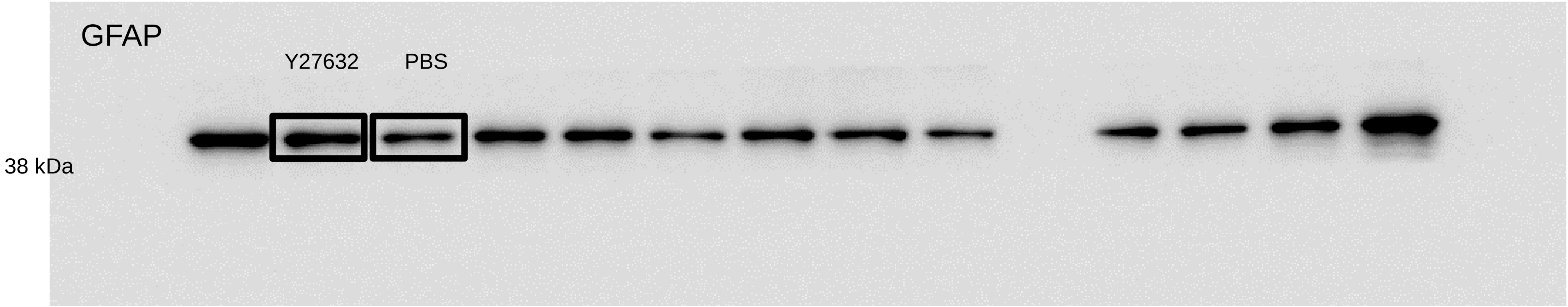

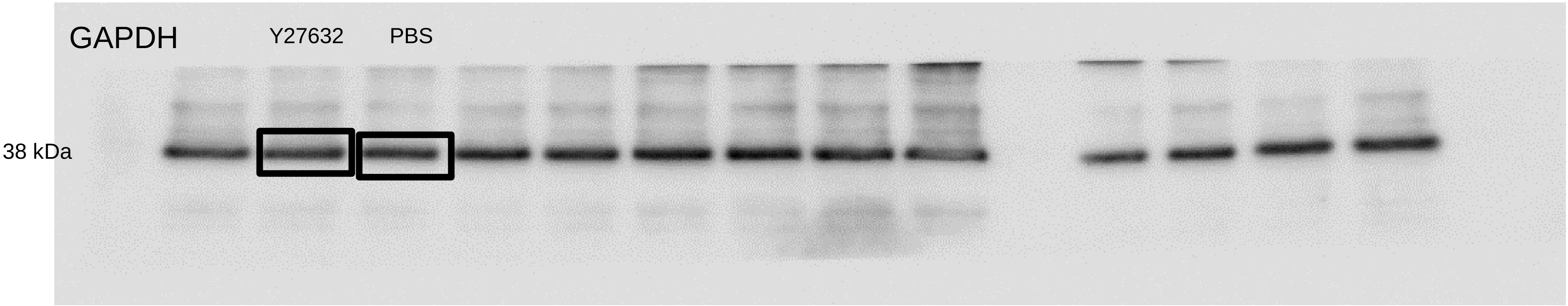

Extended data supplementary figure S4a

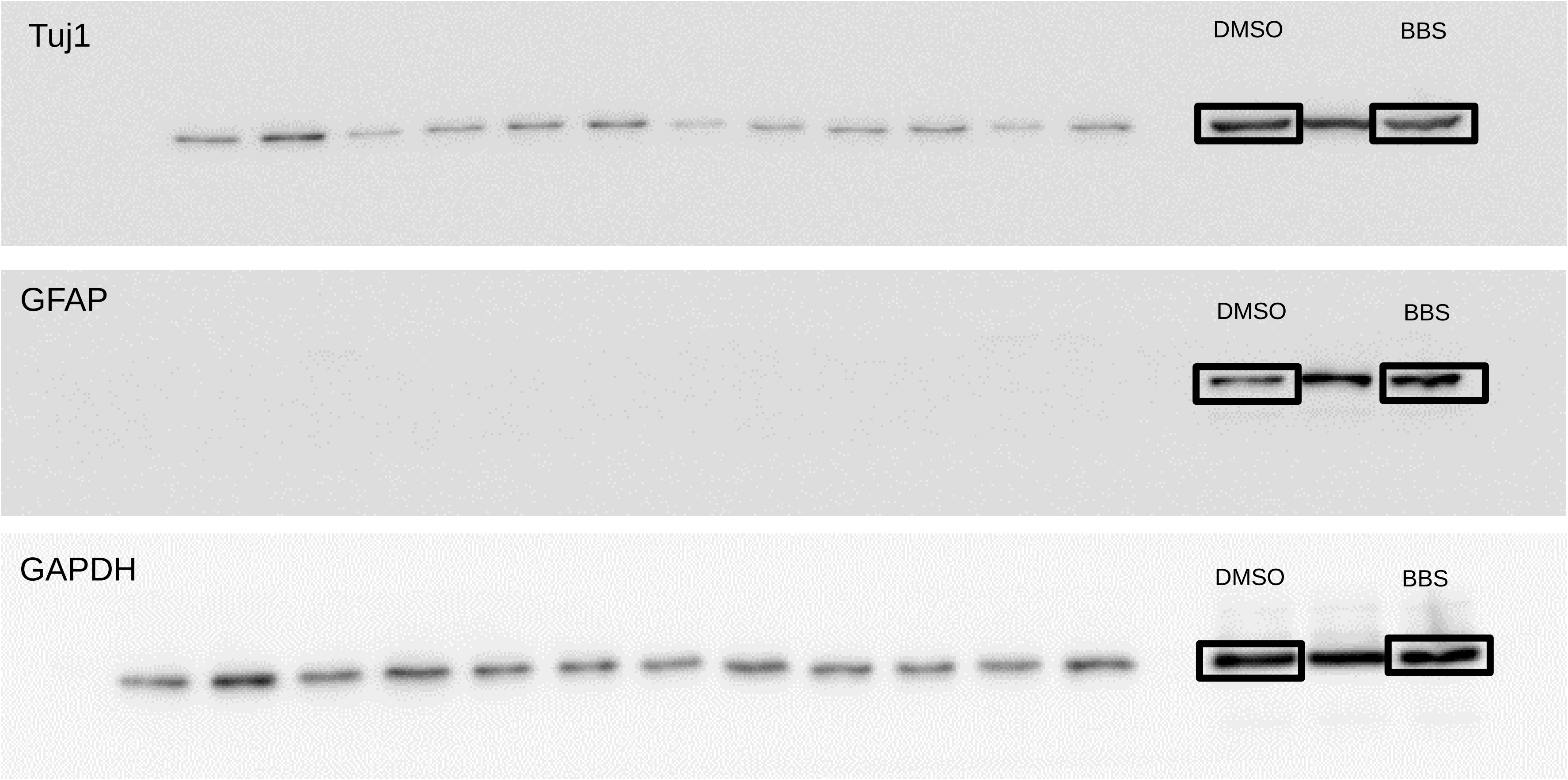

Extended data supplementary figure S5a

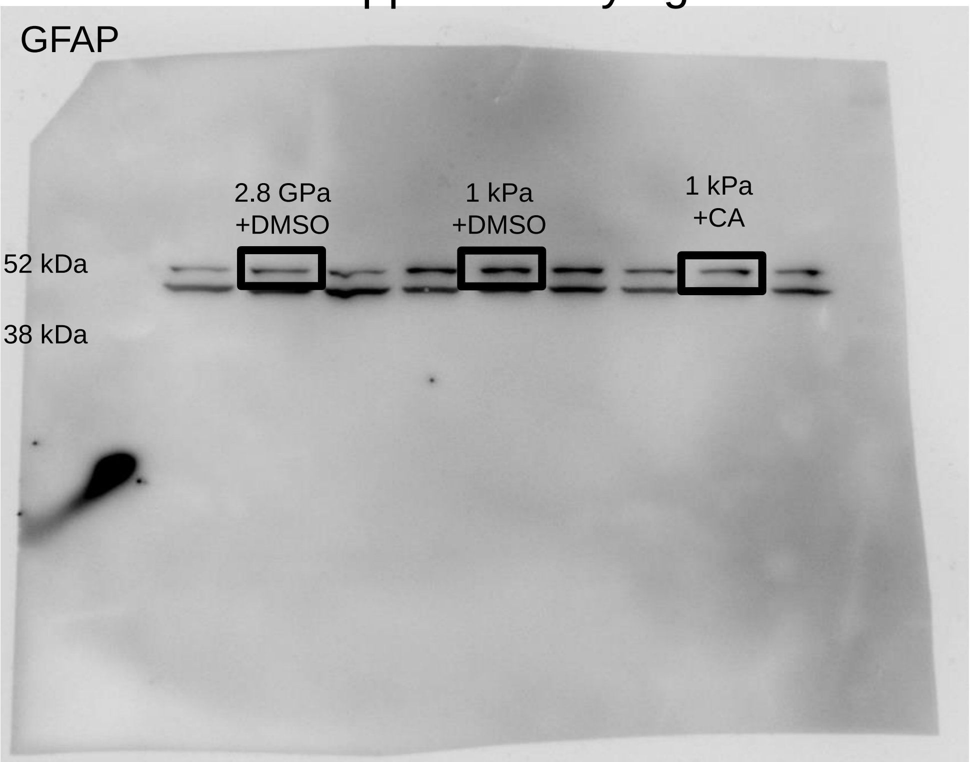

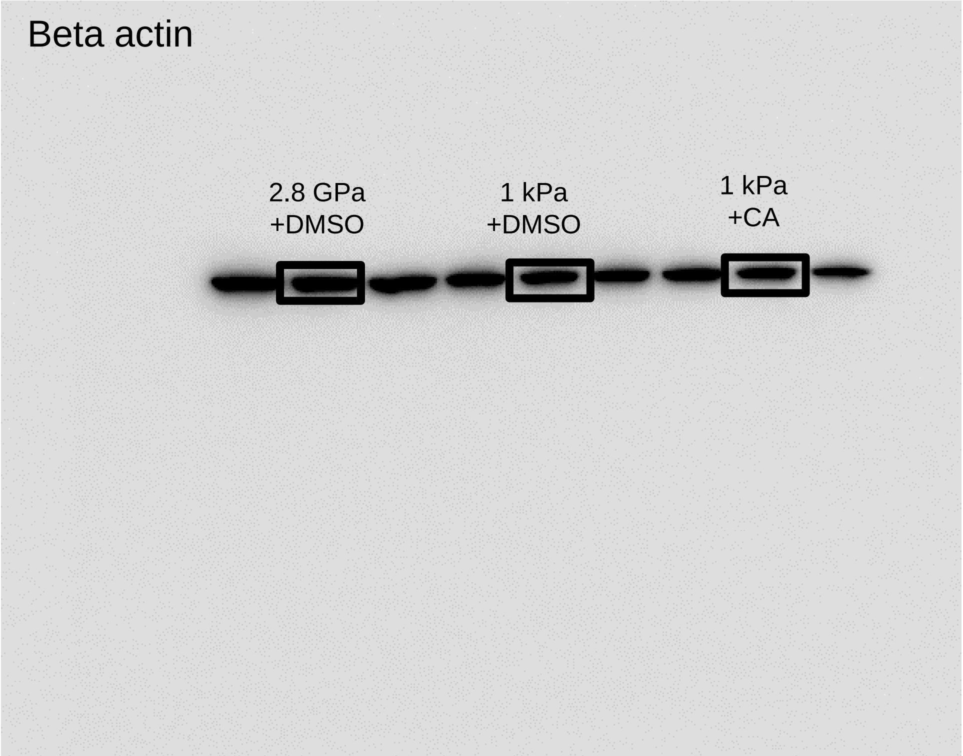

Supplement: Supplementary file 1 — Supplementary Information. [file 41598_2021_99059_MOESM1_ESM.pdf]
